# miR‐AB, a miRNA‐based shRNA viral toolkit for multicolor‐barcoded multiplex RNAi at a single‐cell level

**DOI:** 10.15252/embr.202153691

**Published:** 2022-02-24

**Authors:** Dapeng Wang, Jianbo Xiu, Jiangyue Zhao, Junli Luo

**Affiliations:** ^1^ Department of Immunology Binzhou Medical University Shandong China; ^2^ State Key Laboratory of Medical Molecular Biology Institute of Basic Medical Sciences Chinese Academy of Medical Sciences Beijing China; ^3^ Department of Ophthalmology The 4th Affiliated Hospital of China Medical University Shenyang China; ^4^ Department of Molecular Medicine The Scripps Research Institute Jupiter FL USA

**Keywords:** multicolor, multiplex, RNAi, shRNA, shRNAmir, Methods & Resources, RNA Biology

## Abstract

Uncovering the functions of genes in a complex biological process is fundamental for systems biology. However, currently there is no simple and reliable experimental tool available to conduct loss‐of‐function experiments for multiple genes in every possible combination in a single experiment, which is vital for parsing the interactive role of multiple genes in a given phenotype. In this study, we develop miR‐AB, a new microRNA‐based shRNA (shRNAmir) backbone for simplified, cost‐effective, and error‐proof production of shRNAmirs. After verification of its potent RNAi efficiency *in vitro and in vivo*, miR‐AB was integrated into a viral toolkit containing multiple eukaryotic promoters to enable its application in diverse cell types. We further engineer eight fluorescent proteins emitting wavelengths across the entire visible spectrum into this toolkit and use it to set up a multicolor‐barcoded multiplex RNAi assay where multiple genes are strongly and reliably silenced both individually and combinatorially at a single‐cell level.

## Graphical abstract

A miRNA‐based shRNA viral toolkit allows to set up a multicolor‐barcoded multiplex RNAi assay where multiple genes are silenced in every possible combination at a single cell level.

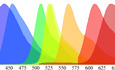

## Introduction

High‐throughput experimental approaches are reasonable ways to attribute a biological consequence to its relevant genes. Among them, multiplex RNAi is a robust genetic approach to perform loss‐of‐function study of multiple target genes. It operates by delivering multiple gene‐specific siRNAs into cells or expressing multiple shRNAs from a multicistronic DNA vector (Silva *et al*, 2008). However, conventional multiplex RNAi assay can only observe the overall phenotype resulted from silencing of all of the target genes, while the contributions of each individual gene or some of them cannot be inferred from the assay. Therefore, development of an RNAi tool that can silence a couple of genes both individually and combinatorially in a single experiment is crucial in an effort to dissect the hierarchical aspects of multiple gene networks, the contributing role of components in a signaling pathway, the functional interdependence of protein complex components, or the functional redundancy or divergence of a gene family.

A multiplex RNAi needs multiple gene‐specific siRNAs or shRNAs in a cell. These excessive siRNAs/shRNAs might affect cell homeostasis because the most considerable limitation of conventional shRNA or siRNA is their interference with endogenous microRNA biogenesis by out‐competing endogenous pre‐microRNA for RISC loading (Snove & Rossi, 2006; Khan *et al*, 2009; Martin *et al*, 2011; van Gestel *et al*, 2014). So, siRNA or conventional shRNA might not be qualified to run a multiplex RNAi. In contrast, microRNA‐based shRNA (shRNAmir) might be a good choice (Zeng *et al*, 2002; Chung *et al*, 2006). These artificial shRNAmirs are processed sequentially by the endonucleases Drosha and Dicer into precisely cleaved siRNAs, similar to the processing of natural microRNAs, thus minimally perturbing cell physiology (Silva *et al*, 2005). Because of their precise cleavage, shRNAmirs also reduce the drawback of off target‐effects of conventional shRNA due to inaccurate processing (Gu *et al*, 2012). Moreover, the use of shRNAmirs can avoid the immune response triggered by regular shRNA expression (Bauer *et al*, 2009). Thanks to the booming field of bioinformatics, two state‐of‐the‐art algorithms, namely, SplashRNA (Pelossof *et al*, 2017) and shERWOOD (Knott *et al*, 2014), were recently developed, which could predict shRNAmirs that effectively and potently silence target genes.

Currently, the most successfully developed and commercialized shRNAmir is based on the human miR‐30 pri‐microRNA backbone, which is further experimentally modified as miR‐E, a variant that shows an enhanced knockdown efficiency. Thus far, a major limitation of miR‐30‐based RNAi is its tedious shRNAmir production, not as simple as conventional shRNA which only needs synthesis of two DNA oligos. Owing to their pri‐microRNA nature, miR‐30‐ or miR‐E‐based shRNAmir cloning relies on PCR (Zuber *et al*, 2011; Fellmann *et al*, 2013; Zhang *et al*, 2020) or Gibson assembly (Rousseaux *et al*, 2018; Michael *et al*, 2019) for *de novo* synthesis of a long DNA sequence (including the terminal loop, upper stem, lower stem, and part of the single‐stranded flanking sequence), which is inserted into the miR‐30 or miR‐E backbone using the endogenous XhoI and EcoRI restriction sites. However, gene‐specific shRNAmirs vary only in the upper stem region, suggesting that a redundant sequence (lower stem + flanking sequence) is included in the current miR‐30‐based cloning strategy.

In this study, a new shRNAmir backbone, named miR‐AB, was developed to simplify the production of shRNAmirs. miR‐AB, in combination of shRNAmirs designed by SplashRNA or shERWOOD, showed remarkable RNAi effects both *in vitro* and *in vivo*. miR‐AB was engineered into a multipromoter viral toolkit carrying eight fluorescent proteins as reporters, which set up a multicolor‐barcoded RNAi assay to efficiently and reliably silence multiple genes both individually and combinatorially at a single‐cell level.

## Results and Discussion

### *De novo* cloning of shRNAmir into miR‐AB is simple, inexpensive, and error‐proof

**Figure 1:**
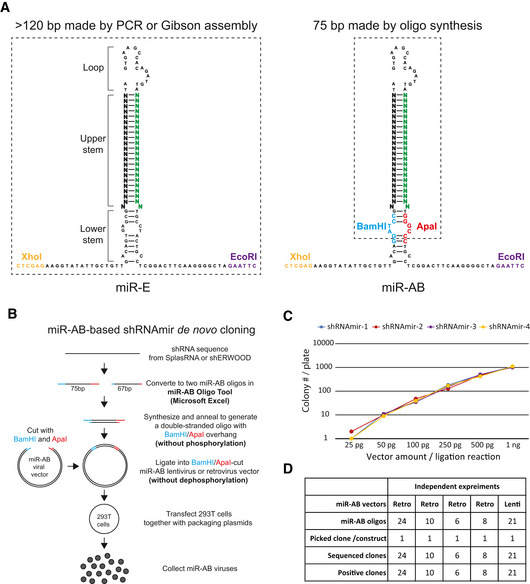
miR‐AB structure and cloning strategy Sequence and structural comparison of miR‐E and miR‐AB. The structures were predicted by CLC Main Workbench. The 22‐bp sequences of passenger/sense (black) and guide/antisense (green) strands are highlighted in large font. The endogenous XhoI and EcoRI restriction sites used for miR‐E cloning and the introduced BamHI and ApaI sites for miR‐AB cloning are colored as indicated. The dashed rectangles indicate the sequences *de novo* generated by PCR or Gibson assembly for miR‐E, or by oligo synthesis with desalt purification for miR‐AB.miR‐AB shRNAmir cloning strategy. A shRNAmir sequence obtained from SplashRNA or shERWOOD platform are converted to two oligos (75 and 67 bp, respectively) by Microsoft Excel‐based miR‐AB Oligo Tool. These two oligos are synthesized, annealed, and ligated to BamHI/ApaI‐cut miR‐AB viral vector for transfection into 293T cells to produce miR‐AB virus. The details of the procedure are described in the Materials and Methods section.miR‐AB cloning efficiency. Various amount of BamHI/ApaI‐cut miR‐AB vectors were ligated with miR‐AB oligos (keep the ratio of vector mass to oligo mole at 1:1; for example, 1 ng of vector with 1 nmol of oligo) and colonies were counted after transformation of XL‐10 Gold competent cells (homemade, 2 × 10^9^ transformation efficiency). The four colored lines represent four individual shRNAmir cloning efficiencies from two independent experiments (two individual shRNAmir cloning in each experiment).miR‐AB cloning reliability. One colony was picked up from each miR‐AB cloning plate for sequencing in five independent experiments using retroviral or lentiviral miR‐AB vectors. The positive clones were calculated.

First, we sought to create a new strategy to simplify the cloning of shRNAmir, which can overcome the shortcoming of current shRNAmir cloning (Fig 1). As mentioned above, cloning of the requisite sequence only (without lower stem flanking sequence) might be the easiest way to accomplish this goal. Since both Drosha (Han *et al*, 2006) and Dicer (Gu *et al*, 2012) cleavages are critically controlled by the shRNA structure but not by its sequence, modification of the shRNAmir backbone by introduction of new restriction sites to the lower stem region may be an efficient strategy. As the Drosha cleavage site is ~11 bp away from the junction of the lower stem and single‐stranded flanking sequence, the forced introduction of new restriction sites will elongate the lower stem and affect the precise cleavage of Drosha. Therefore, replacement of the endogenous sequence of the lower stem with new restriction sites may be a reasonable strategy.

Type IIS restriction enzyme, cleaving DNA outside of its recognition site, is an alternative for cloning of a DNA if appropriate type IIP restriction sites are not available. A previous study demonstrated that BsmBI, a type IIS enzyme, can be used to perform a PCR‐independent cloning of shRNAmir (Adams *et al*, 2017). However, this approach needs phosphorylation of oligos. Moreover, BsmBI is much more expensive than most commonly used type IIP enzymes. Furthermore, type IIS restriction enzymes were reported to show cleavage distance variation or slippage (Lundin *et al*, 2015; Arakawa, 2016), which might result in altered cleavage site leading to low cloning efficiency. So, we tried to introduce the widely used type IIP sites to replace the endogenous sequence. To maximally remove the redundant sequences in *de novo* cloning of gene‐specific shRNAmir, we focused on the lower stem region sequence immediately close to the upper stem (target gene specific) in the miR‐E backbone, screened the available restriction sites that did not conflict with the widely used lentiviral and retroviral systems, and found that ApaI and BamHI sites were good candidates to perform replacement of the endogenous sequence without affecting its spatial structure. After the introduction of these two restriction sites, this new miR‐E variant was named miR‐AB (Fig 1A).

Owing to the existence of these two unique restriction sites, shRNAmir cloning into miR‐AB was simple, as observed in the cloning of a conventional shRNA, wherein two short oligos are synthesized with desalt purification, annealed to generate a DNA duplex with ApaI and BamHI overhangs, and ligated into the ApaI/BamHI‐cut miR‐AB lentiviral or retroviral vector (Fig 1B). To simplify the design of DNA oligos used for miR‐AB cloning, and to efficiently utilize the shRNAmirs designed by the SplashRNA or shERWOOD algorithm, the miR‐AB Oligos Tool, a Microsoft Excel‐based application, was created to convert the 97‐bp gene‐specific shRNA sequence obtained from the SplashRNA or shERWOOD platform to two oligos for *de novo* synthesis. In order to prevent the output error caused by input error, miR‐AB Oligos Tool features an error‐proof design that ensues only 97‐bp sequence can be inputted.

This new approach significantly reduces the cost and time in production of shRNAmir. Specifically, cloning of a shRNAmir by miR‐AB only costs the synthesis of 142 bp oligos (75 + 67 bp), takes 0.5 h for annealing of miR‐AB oligos, and requires minimal labor (mixing and pipetting the oligos), while its cloning by PCR is more cost‐ineffective and labor intensive, including the synthesis of a 97 bp oligo, running a PCR, separating the PCR product on agarose gel, gel purification, and restriction enzyme digestion of the PCR product followed by its purification and quantification.

To test the cloning efficiency of miR‐AB, serial concentration of ApaI/BamHI‐cut retroviral miR‐AB vectors was used to ligate with miR‐AB oligos and the cloning efficiency was determined. As shown in Fig 1C, tens to thousands of colonies were observed on the test plates (no colonies on the miR‐AB vector‐only control plates) and as little as 50 pg of vectors in a ligation generated > 10 colonies, indicating a very high cloning efficiency of miR‐AB. Next, we assessed the reliability of this new shRNAmir cloning strategy. We performed colony screening in multiple independent miR‐AB cloning experiments by picking only one colony from each plate of miR‐AB constructs for DNA sequencing. Strikingly, all the 69 colonies from five independent miR‐AB cloning experiments were 100% positive (Fig 1D). These data demonstrate that miR‐AB can be cloned very efficiently and extremely reliably, which is most useful for shRNAmir library construction.

### miR‐AB shows outstanding RNAi efficiency *in vitro*

**Figure 2:**
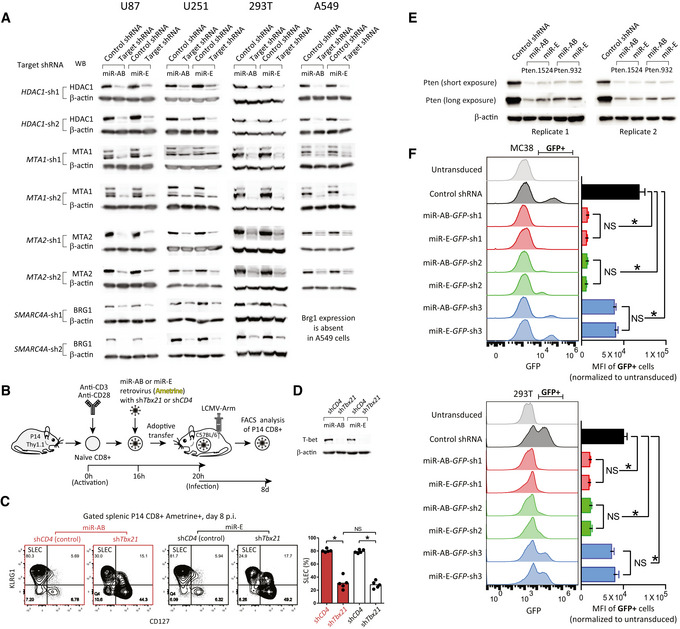
Comparison of RNAi efficiency mediated by miR‐E versus miR‐AB *in vitro* and *in vivo* Two top‐ranked SplashRNA‐designed shRNAmirs targeting human Nurd complex components and a control shRNAmir targeting human *CD4* were cloned into both miR‐AB and miR‐E lentiviral vectors with Venus as reporter. The lentiviruses packaged by these constructs were used for transduction of multiple human cell lines. RNAi efficiency was determined by western blot (WB) analysis of target protein levels in the lysates of the transduced cells. β‐actin served as loading control.A shERWOOD‐designed shRNAmir targeting mouse transcriptional factor *Tbx21* and a control shRNAmir targeting mouse *CD4* were cloned into both miR‐AB and miR‐E retroviral vectors with Ametrine as reporter. The packaged viruses were used for transduction of primary mouse P14 TCR transgenic CD8^+^ T cells, which were used for evaluation of CD8^+^ T cells differentiation *in vivo* as described previously(Wang *et al*, 2018) (see details in the Materials and Methods section).Representative FACS plots of differentiation marker CD127/KLRG1 staining of the gated P14 CD8^+^ T cells and the statistical summary of SLEC subsets were shown to indicate CD8^+^ T cell differentiation. Data are shown as mean ± SD and *n* = 5 mice/group. * indicates significant difference (*P* < 0.01, two‐tailed unpaired Student's *t*‐test). Data are from one representative experiment of two biological replicates.The *Tbx21* RNAi efficiency mediated by miR‐AB vs. miR‐E in the transduced *in vitro*‐expanded P14 CD8^+^ T cells was determined by western blot analysis of T‐bet protein level. β‐actin served as loading control.The RNAi efficiency of Pten.1524 and Pten.932 shRNAmir mediated by miR‐AB versus miR‐E was tested in NIH‐3T3 cells in the context of < 30% transduction efficiency (GFP as reporter). Representative western blots of Pten protein expression of the FACS‐sorted GFP^+^ cells from two biological replicates were shown. β‐actin served as loading control.Three shRNAmirs targeting the GFP reporter of a lentiviral vector in miR‐AB or miR‐E backbone were transduced into MC38 or 293T cells. The RNAi efficiency was determined by FACS analysis of GFP in the context of < 30% transduction efficiency. Data are shown as mean ± SD and *n* = 3 technical replicates. * indicates significant difference (*P* < 0.05). NS indicates no significant difference (*P* > 0.05, two‐tailed unpaired Student's *t*‐test). Source data are available online for this figure.

To determine if miR‐AB possesses the strong RNAi efficiency of miR‐E after the above‐mentioned modifications, we first compared the RNAi efficiency of gene‐specific shRNAmirs in the miR‐AB and miR‐E backbone. Considering the shRNA‐intrinsic variables (McIntyre *et al*, 2011) and differential expression of the RISC complex in different cell types (McFarland *et al*, 2018), it is advisable to assess multiple shRNAmirs targeting multiple genes in multiple cell lines. Therefore, two top‐ranked SplashRNA‐designed shRNAmirs targeting *HDAC1*, *SMARC4A* (encoding BRG‐1), *MTA1*, and *MTA2*, which are components of the Nurd complex ubiquitously expressed in all somatic cells, were cloned into both miR‐AB and miR‐E cassettes in a lentivirus vector. After packaging in 293T cells, viruses were used to infect U87, U251, and A549 human cell lines. A nearly 100% of transduction efficiency was achieved after 72 h of infection. RNAi efficiency was determined by assessing the target protein levels in both the transduced cell lines and the 293T packaging cells. As shown in Fig 2A, a potent RNAi response was triggered by all these gene‐specific shRNAmirs, consistent with the original conclusion that > 90% of the high‐scoring SplashRNA predictions can be considered to markedly silence the expression of target genes (Pelossof *et al*, 2017). Moreover, these observations were cell line independent, further confirming the superiority of these shRNAmirs in the miR‐E and miR‐AB backbone. Importantly, no significant difference in RNAi efficiency was found between miR‐AB and miR‐E for each shRNAmir in the individual cell line, indicating that miR‐AB functioned as effectively as miR‐E. This result was not entirely unexpected because miR‐AB and miR‐E share an identical stem structure which is essential to Drosha cleavage.

### miR‐AB gives a strong RNAi phenotype *in vivo*

Thereafter, in order to comprehensively examine the overall influence of miR‐AB‐based RNAi on target cells, miR‐AB‐mediated RNAi was studied *in vivo* using a CD8^+^ T cell differentiation model (Wang *et al*, 2018). Our previous study revealed that the loss of function of the transcription factor T‐bet, induced by a shERWOOD‐designed and miR‐E‐based shRNAmir, could remarkably suppress the differentiation of short‐lived effector CD8^+^ T cells (SLECs; Wang *et al*, 2018). Based on this finding, CD8^+^ T cell differentiation in the context of miR‐AB‐ or miR‐E‐based RNAi of *Tbx21*, the T‐bet‐encoding gene, was determined *in vivo* (Fig 2B). Consistent with our previous results, the miR‐E‐based control shRNAmir‐expressing P14 CD8^+^ T cells showed a predominant fraction of SLECs, which was the same as that observed in the context of miR‐AB (Fig 2C). In contrast, *Tbx21* shRNAmir expression markedly suppressed SLEC differentiation. Interestingly, this RNAi phenotype was comparable in both the miR‐E and miR‐AB backbones (Fig 2D). To examine if this equivalent *in vivo* phenotype induced by miR‐AB and miR‐E occurred due to their identical RNAi efficacy on the target gene, *Tbx21* RNAi efficiency of these two backbones was determined via western blot analysis of its protein T‐bet in the transduced cells. As expected, miR‐AB‐ and miR‐E‐based *Tbx21* shRNAmir induced almost indistinguishable T‐bet protein loss (Fig 2D), indicating a similar RNAi efficacy of miR‐AB and miR‐E. Together, these data demonstrate that miR‐AB has the same RNAi efficiency as miR‐E *in vivo*.

### miR‐AB holds miR‐E’ single‐copy RNAi efficiency

miR‐E’s advantage over conventional miR‐30 backbone lies in its high RNAi efficiency at a single‐copy level (Fellmann *et al*, 2013). To test if miR‐AB can maintain this advantage, we first determined the RNAi efficiency of Pten.1524 and Pten.932, two shRNAmirs targeting *Pten*, in the backbone of miR‐AB versus miR‐E at a single‐copy level as previously described (Fellmann *et al*, 2013). Consistently, these two shRNAmirs dramatically suppressed the Pten expression in 3T3 cells at < 30% transduction efficiency, an indicative of single‐copy transduction (Fig 2E). Moreover, miR‐AB mediated RNAi as strongly as miR‐E, indicating miR‐AB holds miR‐E’s RNAi potency at a single‐copy level.

To more intuitively show miR‐AB’s RNAi efficiency at a single‐copy level, we carried out a flow cytometry‐based experiment to show transduction efficiency and RNAi efficiency simultaneously. To this end, we cloned three GFP‐specific shRNAmirs into the miR‐AB or miR‐E backbone in a lentiviral vector with a GFP reporter and analyzed their RNAi potency after transduction of MC38 or 293T cells. As shown in Fig 2F, in the context of less than 30% transduction efficiency, these shRNAmirs showed potent (Sh1 and Sh2) or moderate (Sh3) knockdown efficiency (indicated by *) in GFP^+^ cells. Their RNAi potency was comparable between in the miR‐AB versus miR‐E backbone (indicated by NA). It should be noted that the medium‐power Sh3 data are more meaningful to compare miR‐AB versus miR‐E than Sh1 and Sh2, because GFP was almost undetectable in Sh1‐ and Sh2‐transduced cells (their MFI only represents the leftover GFP^+^ cells after RNAi).

### Multiple eukaryotic promoters guarantee miR‐AB‐based RNAi in various cell types

One of the advantages of shRNAmir is its transcription by RNA Pol II (Snyder *et al*, 2009; Fellmann *et al*, 2013). This physiological trait differs dramatically from that of conventional stem‐loop shRNA, which relies on the transcription by RNA Pol III. Unlike RNA Pol III’s relatively constitutive transcription (Dieci *et al*, 2007), RNA Pol II‐directed transcription is tightly regulated, so it is more likely to be silenced in a specific cell type, even in a strong promoter setting. For example, the human cytomegalovirus (CMV) promoter, commonly used in lentiviral vectors, was frequently reported to be silenced in some cell lines (Loser *et al*, 1998; Brooks *et al*, 2004). This might be the underlying reason why some shRNAmirs failed in the induction of a strong RNAi response (Lebbink *et al*, 2011).

To circumvent this caveat and ensure a cell type‐independent RNAi, multiple eukaryotic promoters, such as mouse CMV promoter (Dorsch‐Hasler *et al*, 1985), CBh composite promoter (human CMV enhancer/chicken beta actin promoter/hybrid intron of chicken beta actin intron and minute virus of mice VP intron) (Gray *et al*, 2011), human elongation factor 1 alpha (EF1a) full‐length promoter (Quinn *et al*, 1999) and its derivative, EH composite promoter (human EF1a mini promoter/human leukemia virus’s RU5 region; Attal *et al*, 1996), were introduced to replace the human CMV promoter in the previously established miR‐AB lentiviral vector. Since a few of these promoters contain endogenous restriction sites that can interfere with the cloning of the miR‐AB cassette or vector modification, these recognition sites were modified without changing their cognate universal transcription factor‐binding sites, which were predicted by PROMO (http://alggen.lsi.upc.es/cgi‐bin/promo_v3/promo/promoinit.cgi?dirDB=TF_8.3).

**Figure 3:**
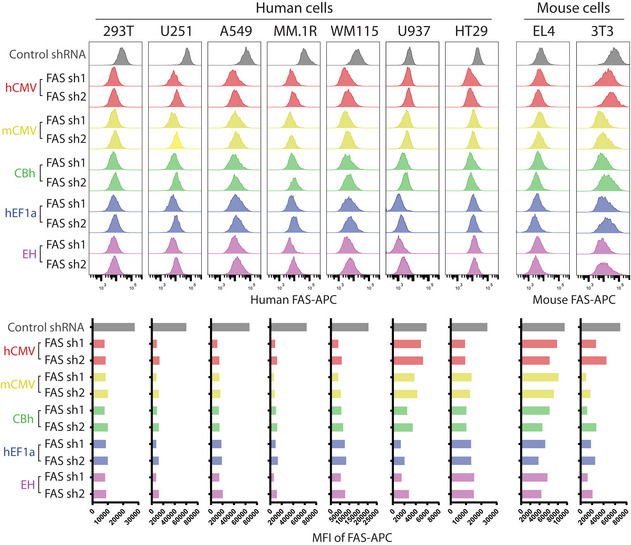
RNAi via miR‐AB driven by multiple eukaryotic promoters in different cell types Two top‐ranked SplashRNA‐designed shRNAmirs targeting human or mouse surface protein *FAS* were cloned into Venus‐expressing miR‐AB lentiviral vectors which harbor different promoters to drive the shRNAmir expression. The packaged lentiviruses were used to infect the indicated human or mouse cell lines. A shRNAmir targeting human or mouse *CD4* driven under hCMV promoter was used as control. After > 96 h of infection, FAS protein levels in the transduced Venus‐positive cells were determined by surface staining, shown in histograms (top panels) and quantified by MFI of APC (bottom panels).

To test whether these promoters could function properly in these miR‐AB lentiviral vectors, two top‐ranked SplashRNA‐designed shRNAmirs targeting the human or mouse *FAS* receptor, a surface protein that is constitutively expressed in most tissues (Peter *et al*, 2007), were cloned into miR‐AB lentiviral vectors harboring these promoters. These new constructs were packaged in 293T cells as effectively as the original vector with the human CMV promoter and maintained the intensity of the Venus reporter. A cost‐effective transfection of 293T cells with these constructs by PEI (~40% transfection efficiency) can generate 3–5 × 10^6^ transduction unit titers of virus from one well of a six‐well plate. Commercial transfection reagent can generate higher titers by increasing transfection efficiency. For a precise determination of RNAi efficiency of these new constructs, FAS protein levels were assessed via flow cytometry using multiple human and mouse cell lines. Consistent with the above‐mentioned data, all shRNAmirs effectively silenced FAS protein expression (Fig 3), further confirming the predictive efficiency of this algorithm.

In most cell lines, these shRNAmirs exhibited similar and remarkable RNAi effects, irrespective of the promoter type. However, they showed differential RNAi results in certain cell lines (Fig 3). For instance, human CMV promoter induced a poor RNAi response in both human U937 cells and mouse NIH‐3T3 cells (Fig 3). Since human CMV promoter had a weak transcriptional activity in mouse cells (Addison *et al*, 1997), it is not surprising that it failed to transcribe enough miR‐AB shRNAmirs to strongly silence target genes in mouse NIH‐3T3 cells. However, it is unexpected that human CMV promoter‐directed miR‐AB did not exhibit a strong RNAi response in U937 cells, indicating the cell type‐specific variation of these promoters’ activities. This cell line‐specific uncertainty was the driving force to develop the multipromoter miR‐AB RNAi vectors in this study. The multipromoter design can maximally solve the problems frequently found in RNAi experiments.

### Construction of a multipromoter and multicolor miR‐AB‐based viral toolkit

**Figure 4:**
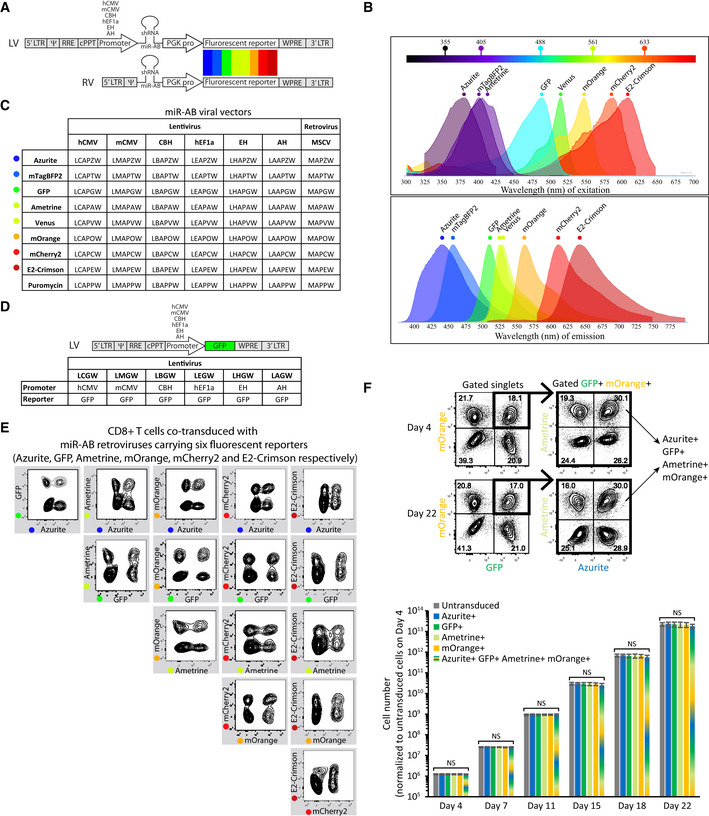
miR‐AB‐based multipromoter and multicolor viral toolkit Schematic diagram of miR‐AB‐based lentiviral (LV) and retroviral (RV) vectors. miR‐AB shRNAmir expression is driven by a composite promoter composed of the endogenous 5′ LTR of HIV and a eukaryotic promoter in lentiviral vectors, or by the endogenous 5′ LTR of MSCV in retroviral vectors. Eight fluorescent proteins with emission peaks across whole visible spectrum are controlled by mouse PGK promoter. A woodchuck hepatitis post‐transcriptional regulatory element (WPRE) is used to enhance transgene expression.The excitation and emission spectra of these fluorescent proteins are shown in FPbase Spectra Viewer (https://www.fpbase.org/spectra/). The five commonly used lasers are indicated in the spectrum. The excitation spectrum of Azurite is shown as that of its parental protein EBFP due to lack of information in the database.Nomenclature of 56 miR‐AB‐based lentiviral and retroviral vectors with different promoters and different fluorescent protein reporters. AH promoter (human beta actin promoter (Ng *et al*, 1989)/human leukemia viruses RU5 region)‐based miR‐AB lentiviral vectors, the most recently developed one, also exhibited strong RNAi efficiency. Puromycin was also engineered into this toolkit for antibiotic‐based screening of transformants.Lentiviral vectors with GFP reporter that are controlled by the promoters used in miR‐AB lentiviral vectors were constructed for easy assessment of the promoters activity on microscope or flow cytometer.CD8^+^ T cells were activated and then cotransduced with miR‐AB retroviruses targeting *CD4* with Azurite, GFP, Ametrine, mOrange, mCherry2, or E2‐Crimson as fluorescent reporters. The fluorescence of all fluorescent proteins was plotted on contour plots showing the fluorescent separation of each pair of the fluorescent proteins. The data are representative of at least three biologically independent experiments.293T cells were cotransduced with four miR‐AB lentiviruses at 1:1 ratio, which carries a neutral shRNAmir targeting *CD4* or *CD19*, with Azurite, GFP, Ametrine, or mOrange as reporter, respectively. Cells were cultured for 22 days and fluorescence‐positive cells were quantified at the time points as indicated. Representative contour FACS plots displaying the fluorescent reporter^+^ cells on day 4 and day 22 are shown (top panels). Cell numbers of untransduced, single fluorescence‐positive cells, and quadruple positive cells were quantified and normalized to the untransduced cells on day 4, and plotted over time (bottom panel). Data are shown as mean ± SD and *n* = 4 technical replicates. NS indicates no significant difference (*P* > 0.05, two‐tailed unpaired Student's *t*‐test).

In order to construct a novel RNAi tool, eight fluorescent proteins with emission wavelengths spanning the entire visible spectrum were engineered into the above‐established lentiviral and retroviral miR‐AB vectors to constitute a multipromoter and multicolour RNAi viral toolkit (Fig 4A–C). To facilitate the proper choice of these promoters, six lentiviral vectors with GFP as reporter that is controlled by different promoters were constructed to simply assess the promoter activity on a microscope or flow cytometer (Fig 4D). The fluorescent proteins in this toolkit can be readily detected by most widely used flow cytometers or fluorescent microscopes without need of changing parameters (Table 1). Since the original coding sequences of these fluorescent proteins contain a couple of restriction sites that are detrimental to their cloning into a given plasmid, most of these restriction sites, especially the ones that are frequently found in popular plasmids, were removed by codon optimization. Most of these fluorescent proteins have an emission peak that is quite different from the others. Ametrine, a distinctive GFP derivative, has a long Stoke’ shift that makes it compatible with GFP and Venus irrespective of their overlapping emission spectra (Ai *et al*, 2008). These characteristics make these fluorescent proteins suitable for construction of a multicolor panel to simultaneously visualize multiple cell populations with different genotypes. Indeed, six fluorescent reporters including Azurite, GFP, Ametrine, mOrange, mCherry2, and E2‐Crimson set up a multicolor panel in CD8^+^ T cells where every two fluorescent reporters emitted fluorescence that were widely separated from each other, showing a clear four‐quadrant plot (Fig 4E). This indicated these fluorescent reporter vectors can generate sufficiently high fluorescent signals that are easily detected and distinguished by flow cytometer. It should be noted that this flow cytometry analysis was conducted on BD FACSAria™ III Cell Sorter, a widely used flow cytometer that is equipped with four standard lasers with 405, 488, 561, and 633 nm excitations, which are not the optimal excitation wavelengths of some of the fluorescent proteins used in this experiment. Therefore, optimal laser‐installed flow cytometers or fluorescent microscopes could maximally take advantage of these fluorescent reporters in a multicolor assay.

**Table 1:** Flow cytometry parameters of a five lasers‐equipped FACSAria™ III flow cytometer for detection of the fluorescent proteins.

	Laser	Band pass filter	Commonly used fluorophores with similar spectral characteristics
Azurite	UV (355 nm)	450/40	Indo‐1 hi, Alexa Fluor 350, Marina Blue
	Violet (405 nm)	450/40	Brilliant Violet 421, Pacific Blue, Alexa Fluor 405, Sytox Blue
mTagBFP2	Violet (405 nm)	450/40	Brilliant Violet 421, Pacific Blue, Alexa Fluor 405, Sytox Blue
Ametrine	Violet (405 nm)	510/50	Brilliant Violet 510, AmCyan, Krome Orange
EGFP	Blue (488 nm)	530/30	FITC, Alexa Fluor 488, CFSE, Sytox Green, Dylight 488
Venus	Blue (488 nm)	530/30	FITC, Alexa Fluor 488, CFSE, Sytox Green, Dylight 488
mOrange	Blue (488 nm) or Yellow/Green (561 nm)	582/15	PE, PI, Alexa Fluor 555, Dylight 549
mCherry2	Yellow/Green (561 nm)	610/20	PE/Texas Red, Alexa Fluor 594, PE‐CF594, Dylight 594
E2‐Crimson	Red (633 nm)	660/20	APC, Alexa Fuor 647, Cy5, Sytox Red, Dylight 649

These fluorescent proteins were deliberately chosen according to not only their excitation and emission wavelength peaks but also their other characteristics, such as good photo stability and chromophore chemical stability, high fluorescent intensity, and fast maturation. In addition, Aequorea Victoria‐derived fluorescent proteins (GFP, Azurite, Ametrine and Venus) and mOrange were included in this toolkit as they showed low immunogenicity (Gossa *et al*, 2014; Wang *et al*, 2018), making them especially useful for performing adoptive transfer experiments in mice. To expand the application of miR‐AB, codon‐optimized puromycin resistance gene was integrated into this RNAi system for selection and maintenance of cells expressing shRNAmirs (Fig 4C).

### Multiple shRNAmir transduction is not cytotoxic

The aim of this study was to develop a novel multiplex RNAi assay. We chose shRNAmir but not conventional shRNA in this assay because shRNAmir displayed minimal cytotoxicity. Indeed, our experimental data showed the cotransduction of 293T cells with four viruses carrying a neutral shRNAmir did not significantly alter the four fluorescent reporters expressing cell expansion after 22 days of culture (Fig 4F). Since the multiple shRNAmirs‐expressing cells always account for a small portion of the whole cell population (< 10% in this experiment), like a low‐efficiency transduction of a single shRNAmir, it is very likely that they express each shRNAmir at a single‐copy level. So, their total shRNAmirs level will not be super abundant. Given the advantage of shRNAmir over conventional shRNA in maintaining cell homeostasis, multiple shRNAmirs transduction might not be problematic.

### Multicolor‐barcoded multiplex RNAi efficiently and reliably silences multiple target genes both individually and combinatorially at a single‐cell level

**Figure 5:**
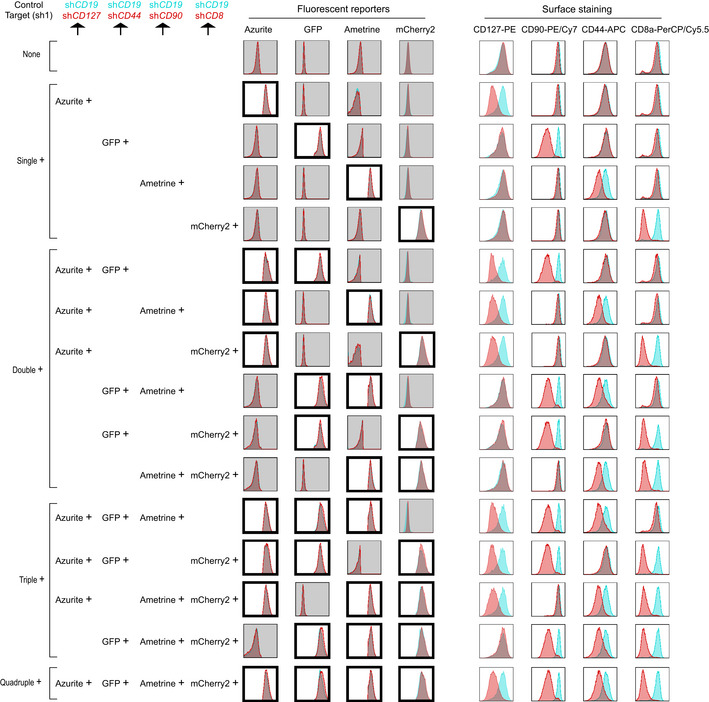
miR‐AB‐based multicolor‐barcoded multiplex RNAi CD8^+^ T cells were activated for 16 h and then cotransduced with miR‐AB retroviruses at 1:1 ratio, which carries a SplashRNA‐designed shRNAmir, targeting surface protein *CD127, CD90, CD44, or CD8*, with Azurite, GFP, Ametrine, or mCherry2 as fluorescent reporters, respectively, for 4 h. miR‐AB retroviruses expressing *CD19*‐specific shRNAmirs with same fluorescent reporters were used as controls. Surface staining was performed 72 h after transduction. FACS data were shown as overlaid histograms of control shRNAmirs (sh*CD19*s) and target shRNAmirs (sh*CD127*, sh*CD90*, sh*CD44*, or sh*CD8*). Cells were categorized as 16 populations expressing single, double, triple, or quadruple fluorescent proteins or not (none) and fluorescent reporter‐positive plots were highlighted with thick frames and white background. Each population’s surface staining profile was shown on the right. The data are representative of two biologically independent experiments.

**Figure EV1:**
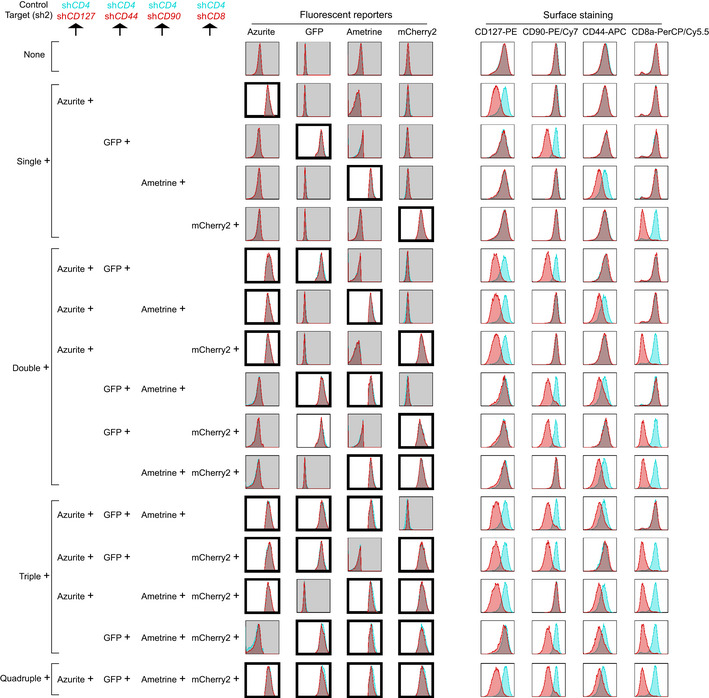
Multicolor‐barcoded multiplex RNAi by miR‐AB retrovirus in CD8^+^ T cells This RNAi experiment was carried out as described in Fig 5 but used another SplashRNA‐designed shRNAmirs targeting *CD127, CD90, CD44, or CD8. CD4*‐specific shRNAmirs were used as controls. The data are representative of two biologically independent experiments.

**Figure EV2:**
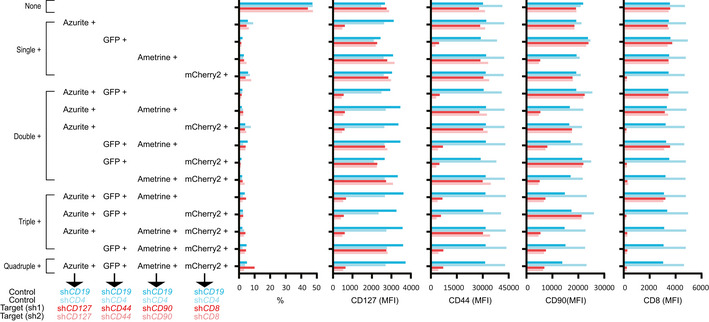
RNAi efficiency quantification of FACS plots in Figs 5 and EV1 Target gene expression was quantified by calculating its MFI in FACS plots. The percentage of all fluorescence‐positive cells (untransduced, single, double, triple, and quadruple) were also shown (first column panels).

Finally, we tried to use this toolkit to set up a multicolor‐barcoded multiplex RNAi assay where loss‐of‐function effects of target genes could be analyzed at both single‐gene level and multiple‐gene level. To this end, four CD8^+^ T cell surface proteins, CD127, CD90, CD44, and CD8a were knocked down in mouse CD8^+^ T cells by cotransduction of miR‐AB retroviruses expressing either of the two top‐ranked SplashRNA‐designed shRNAmirs targeting *CD127, CD90, CD44, or CD8a* with Azurite, GFP, Ametrine, and mCherry2 as fluorescent reporters, respectively (Fig 5 and EV1). In parallel, similar *CD19* (Fig 5) or *CD4* (Fig EV1) RNAi was used as control. Obviously, the cotransduced cells showed 16 cell populations which expressed different combinations of the 4 fluorescent reporters. The overall percentage of these populations was > 50% (untransduced cell percentage was <50%, top bars in Fig EV2), indicating these viruses had high viral titers because primary CD8^+^ T cells are harder to be infected than cell lines. The lowest individual percentage of these population was > 1%, indicating there are enough cells for FACS analysis of each population in this assay (hundreds of cells can give a clear FACS population).

**Table 2:** The list of miR‐AB oligos used for cloning of shRNAmirs in this study.

Target gene	shRNAmir	Species	Designed by	miR‐AB oligos
HDAC1	sh1	Human	SplashRNA	GATCCGAGGTTAGGTTGCTTCAATCTAATAGTGAAGCCACAGATGTATTAGATTGAAGCAACCTAACCGTGGGCC
				CACGGTTAGGTTGCTTCAATCTAATACATCTGTGGCTTCACTATTAGATTGAAGCAACCTAACCTCG
HDAC1	sh2	Human	SplashRNA	GATCCGACACAGCGATGACTACATTAAATAGTGAAGCCACAGATGTATTTAATGTAGTCATCGCTGTGGTGGGCC
				CACCACAGCGATGACTACATTAAATACATCTGTGGCTTCACTATTTAATGTAGTCATCGCTGTGTCG
SMAR4A	sh1	Human	SplashRNA	GATCCGATGGATGTCAAACAGTAATAAATAGTGAAGCCACAGATGTATTTATTACTGTTTGACATCCAGTGGGCC
				CACTGGATGTCAAACAGTAATAAATACATCTGTGGCTTCACTATTTATTACTGTTTGACATCCATCG
SMAR4A	sh2	Human	SplashRNA	GATCCGACCGTGGACTTCAAGAAGATAATAGTGAAGCCACAGATGTATTATCTTCTTGAAGTCCACGGGTGGGCC
				CACCCGTGGACTTCAAGAAGATAATACATCTGTGGCTTCACTATTATCTTCTTGAAGTCCACGGTCG
MTA1	sh1	Human	SplashRNA	GATCCGCTCAAGTAATTTTCATATTAAATAGTGAAGCCACAGATGTATTTAATATGAAAATTACTTGAATGGGCC
				CATTCAAGTAATTTTCATATTAAATACATCTGTGGCTTCACTATTTAATATGAAAATTACTTGAGCG
MTA1	sh2	Human	SplashRNA	GATCCGAAAGGTGCCATTTTAAATTTTATAGTGAAGCCACAGATGTATAAAATTTAAAATGGCACCTTCTGGGCC
				CAGAAGGTGCCATTTTAAATTTTATACATCTGTGGCTTCACTATAAAATTTAAAATGGCACCTTTCG
MTA2	sh1	Human	SplashRNA	GATCCGACAGCATAGTCCAGTTTTATTATAGTGAAGCCACAGATGTATAATAAAACTGGACTATGCTGGTGGGCC
				CACCAGCATAGTCCAGTTTTATTATACATCTGTGGCTTCACTATAATAAAACTGGACTATGCTGTCG
MTA2	sh2	Human	SplashRNA	GATCCGCACGCCAGTTCTCAGAAATAAATAGTGAAGCCACAGATGTATTTATTTCTGAGAACTGGCGTATGGGCC
				CATACGCCAGTTCTCAGAAATAAATACATCTGTGGCTTCACTATTTATTTCTGAGAACTGGCGTGCG
FAS	sh1	Human	SplashRNA	GATCCGCAGTGTTTGAAAAGATTCTTAATAGTGAAGCCACAGATGTATTAAGAATCTTTTCAAACACTATGGGCC
				CATAGTGTTTGAAAAGATTCTTAATACATCTGTGGCTTCACTATTAAGAATCTTTTCAAACACTGCG
FAS	sh2	Human	SplashRNA	GATCCGATCCAAGGATGTTTAAAATCTATAGTGAAGCCACAGATGTATAGATTTTAAACATCCTTGGAGTGGGCC
				CACTCCAAGGATGTTTAAAATCTATACATCTGTGGCTTCACTATAGATTTTAAACATCCTTGGATCG
CD4	sh1	Human	SplashRNA	GATCCGACCTGATCATCAAGAATCTTAATAGTGAAGCCACAGATGTATTAAGATTCTTGATGATCAGGGTGGGCC
				CACCCTGATCATCAAGAATCTTAATACATCTGTGGCTTCACTATTAAGATTCTTGATGATCAGGTCG
FAS	sh1	Mouse	SplashRNA	GATCCGAACAGTTAAGAGTTCATACTCATAGTGAAGCCACAGATGTATGAGTATGAACTCTTAACTGTGTGGGCC
				CACACAGTTAAGAGTTCATACTCATACATCTGTGGCTTCACTATGAGTATGAACTCTTAACTGTTCG
FAS	sh2	Mouse	SplashRNA	GATCCGACGGGTTCGTGAAACTGATAAATAGTGAAGCCACAGATGTATTTATCAGTTTCACGAACCCGCTGGGCC
				CAGCGGGTTCGTGAAACTGATAAATACATCTGTGGCTTCACTATTTATCAGTTTCACGAACCCGTCG
Tbx21	sh1	Mouse	shERWOOD	GATCCGCCACACACGTCTTTACTTTCCATAGTGAAGCCACAGATGTATGGAAAGTAAAGACGTGTGTGTTGGGCC
				CAACACACACGTCTTTACTTTCCATACATCTGTGGCTTCACTATGGAAAGTAAAGACGTGTGTGGCG
CD4	sh1	Mouse	shERWOOD	GATCCGAGCATGGGAGAAAGGATCGTTTTAGTGAAGCCACAGATGTAAAACGATCCTTTCTCCCATGCCTGGGCC
				CAGGCATGGGAGAAAGGATCGTTTTACATCTGTGGCTTCACTAAAACGATCCTTTCTCCCATGCTCG
CD4	sh1	Mouse	SplashRNA	GATCCGCACAGCATATCTTAATTCATAATAGTGAAGCCACAGATGTATTATGAATTAAGATATGCTGTTTGGGCC
				CAAACAGCATATCTTAATTCATAATACATCTGTGGCTTCACTATTATGAATTAAGATATGCTGTGCG
CD19	sh1	Mouse	SplashRNA	GATCCGACAGTCCTATGAAGATATGAGATAGTGAAGCCACAGATGTATCTCATATCTTCATAGGACTGGTGGGCC
				CACCAGTCCTATGAAGATATGAGATACATCTGTGGCTTCACTATCTCATATCTTCATAGGACTGTCG
CD127	sh1	Mouse	SplashRNA	GATCCGCGGGTAAGTTATTCAAATTCAATAGTGAAGCCACAGATGTATTGAATTTGAATAACTTACCCATGGGCC
				CATGGGTAAGTTATTCAAATTCAATACATCTGTGGCTTCACTATTGAATTTGAATAACTTACCCGCG
CD127	sh2	Mouse	SplashRNA	GATCCGCCCATGTCTAGTTTTTACCAAATAGTGAAGCCACAGATGTATTTGGTAAAAACTAGACATGGTTGGGCC
				CAACCATGTCTAGTTTTTACCAAATACATCTGTGGCTTCACTATTTGGTAAAAACTAGACATGGGCG
CD44	sh1	Mouse	SplashRNA	GATCCGCCAGGTTTGAGTTTATATCAAATAGTGAAGCCACAGATGTATTTGATATAAACTCAAACCTGATGGGCC
				CATCAGGTTTGAGTTTATATCAAATACATCTGTGGCTTCACTATTTGATATAAACTCAAACCTGGCG
CD44	sh2	Mouse	SplashRNA	GATCCGAAGGGTATAAATTGATTCATAATAGTGAAGCCACAGATGTATTATGAATCAATTTATACCCTGTGGGCC
				CACAGGGTATAAATTGATTCATAATACATCTGTGGCTTCACTATTATGAATCAATTTATACCCTTCG
CD90	sh1	Mouse	SplashRNA	GATCCGAAGGGCTGCTTCTGATTATTTATAGTGAAGCCACAGATGTATAAATAATCAGAAGCAGCCCTGTGGGCC
				CACAGGGCTGCTTCTGATTATTTATACATCTGTGGCTTCACTATAAATAATCAGAAGCAGCCCTTCG
CD90	sh2	Mouse	SplashRNA	GATCCGCGCTGTCATTTTGTACTCTGTATAGTGAAGCCACAGATGTATACAGAGTACAAAATGACAGCTTGGGCC
				CAAGCTGTCATTTTGTACTCTGTATACATCTGTGGCTTCACTATACAGAGTACAAAATGACAGCGCG
CD8	sh1	Mouse	SplashRNA	GATCCGCCAGTTCCTTTTTCTTTATGAATAGTGAAGCCACAGATGTATTCATAAAGAAAAAGGAACTGTTGGGCC
				CAACAGTTCCTTTTTCTTTATGAATACATCTGTGGCTTCACTATTCATAAAGAAAAAGGAACTGGCG
CD8	sh2	Mouse	SplashRNA	GATCCGACTGTAGTAGAATCCAATTAAATAGTGAAGCCACAGATGTATTTAATTGGATTCTACTACAGCTGGGCC
				CAGCTGTAGTAGAATCCAATTAAATACATCTGTGGCTTCACTATTTAATTGGATTCTACTACAGTCG
Pten	1524	Mouse	SplashRNA	GATCCGACAGCTAAAGGTGAAGATATATTAGTGAAGCCACAGATGTAATATATCTTCACCTTTAGCTGGTGGGCC
				CACCAGCTAAAGGTGAAGATATATTACATCTGTGGCTTCACTAATATATCTTCACCTTTAGCTGTCG
Pten	932	Mouse	SplashRNA	GATCCGCCGACTTAGACTTGACCTATATTAGTGAAGCCACAGATGTAATATAGGTCAAGTCTAAGTCGATGGGCC
				CATCGACTTAGACTTGACCTATATTACATCTGTGGCTTCACTAATATAGGTCAAGTCTAAGTCGGCG
GFP	sh1	N/A	SplashRNA	GATCCGAATGGACGAGCTGTACAAGTAATAGTGAAGCCACAGATGTATTACTTGTACAGCTCGTCCATGTGGGCC
				CACATGGACGAGCTGTACAAGTAATACATCTGTGGCTTCACTATTACTTGTACAGCTCGTCCATTCG
GFP	sh2	N/A	SplashRNA	GATCCGAACAAGCTGGAGTACAACTACATAGTGAAGCCACAGATGTATGTAGTTGTACTCCAGCTTGTGTGGGCC
				CACACAAGCTGGAGTACAACTACATACATCTGTGGCTTCACTATGTAGTTGTACTCCAGCTTGTTCG
GFP	sh3	N/A	SplashRNA	GATCCGCCAAGCAGAAGAACGGCATCAATAGTGAAGCCACAGATGTATTGATGCCGTTCTTCTGCTTGTTGGGCC
				CAACAAGCAGAAGAACGGCATCAATACATCTGTGGCTTCACTATTGATGCCGTTCTTCTGCTTGGCG

Then we analyzed the RNAi profile of all the 16 cell populations. As expected, all of the fluorescent reporter single‐positive cells (Single^+^ panels in Figs 5, EV1 and EV2) showed dramatic RNAi effects of the target gene‐specific shRNAmir as compared with control cells (sh*CD19* in Fig 5 and sh*CD4* in Fig EV1), while the expression of the other three nontarget genes was not affected. Next, we checked the double RNAi effects by looking at the fluorescent reporter double‐positive cells. There were six cell populations that expressed only two of the four fluorescent reporters (Double^+^ panels in Figs 5 and EV1). Each population displayed a distinct surface staining profile that were completely related to the two fluorescent reporters of target genes RNAi. Similarly, triple RNAi were also successfully and faithfully achieved (Triple^+^ panels in Figs 5 and EV1 and EV2). Quadruple RNAi knocked down all the four target genes, resembling a conventional multiplex RNAi of four genes (Quadruple^+^ panels in Figs 5 and EV1 and EV2). Importantly, the RNAi efficiency of each target gene‐specific shRNAmir was almost identical among its fluorescent reporter‐positive cells in single^+^, double^+^, triple^+^, and quadruple^+^ cell populations (MFI in Fig EV2) and all of the fluorescent reporter‐negative cell populations showed indistinguishable normal expression of target genes, indicating the reliability and unbiasedness of the fluorescent reporters in this multiplex RNAi assay. In summary, this multicolor‐barcoded miR‐AB‐based multiplex RNAi assay strongly and reliably recorded both single‐gene and multiple‐gene RNAi at a single‐cell level.

### Guidance of Multicolor‐barcoded multiplex RNAi

In theory, all of the eight fluorescent reporters can be distinguished from each other if optimal lasers and bandpass filters are available. The selection of fluorescent reporters is not only dependent on the fluorescent characteristics of them but also on the availability and performance of the phenotypic marker antibody whose conjugated fluorophore must be distinguishable from the fluorescent reporters used. For instance, in our four color‐barcoded RNAi experiment including *CD127* RNAi (Fig 5 and EV1), mOrange is not selected because it is indistinguishable from PE, which is the fluorophore of PE anti‐mouse CD127, a best fluorophore‐conjugated CD127 antibody available. Another important aspect of setting up a reliable multicolor RNAi assay is how and where to test the RNAi efficiency. The most persuasive way to determine the knockdown efficiency is an “on‐site” test by staining the color‐barcoded cells with the fluorophore‐conjugated target gene‐specific antibodies (Figs 5 and EV1). However, if this “on‐site” test is not applicable (such as due to limited color choice when plenty of phenotyping markers are being tested), RNAi response can be determined elsewhere (such as another independent flow cytometry experiment). This less‐than‐ideal alternative is credible because our data demonstrated unbiased RNAi efficiency of target genes in target gene‐specific shRNAmir‐expressing cells, irrespective of co‐expression of other gene‐specific shRNAmirs or not (Figs 5 and EV1 and EV2). If target gene‐specific flow cytometry antibody or immunohistochemistry antibody is unavailable, multicolor‐barcoded cells can be sorted out for RNAi verification or phenotyping analysis.

**Figure EV3:**
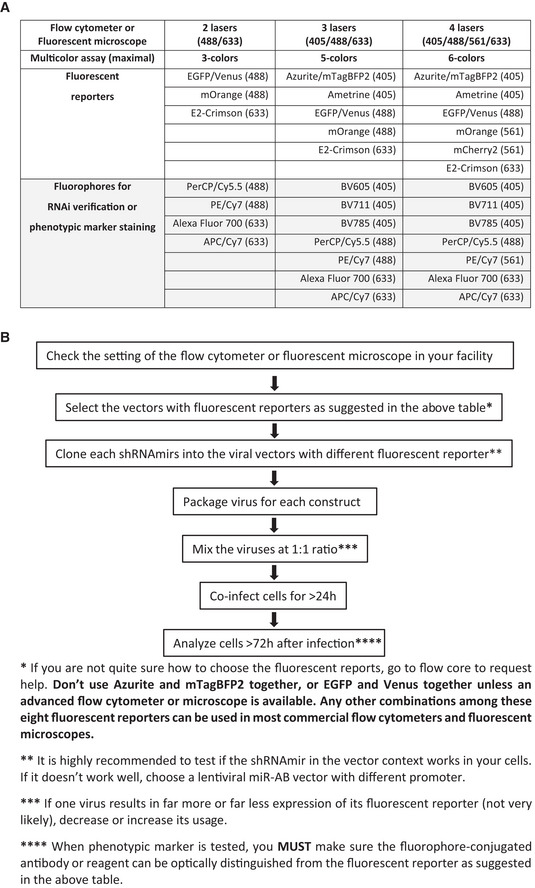
Guidance of setting up a multicolor‐barcoded multiplex RNAi Multicolor‐barcoded multiplex RNAi setup guidance. The fluorescent reporters for the RNAi assay and the fluorophores for RNAi verification or phenotypic marker staining are dependent on the lasers on a flow cytometer or fluorescent microscope. The optimal laser wavelength is indicated for each fluorescent reporter or fluorophore in a multicolor assay.A flowchart of performing a multicolor‐barcoded RNAi experiment. Some suggestions and recommendations are listed below.

To help easily set up a proper multicolor‐barcoded multiplex RNAi assay, some suggestions and recommendations were described in Fig EV3A and B to instruct how to choose fluorescent reporters and fluorophores for RNAi verification or phenotypic marker staining on the most widely available flow cytometers with two, three, or four lasers. We especially highlighted the caveat that “Don’t use Azurite and mTagBFP2 together, or GFP and Venus together unless an advanced flow cytometer or microscope is available. Any other combinations among these eight fluorescent reporters can be used in most commercial flow cytometers and fluorescent microscopes”. We also drew a flowchart of performing a multicolor‐based multiplex RNAi assay for avoiding mistakes (Fig EV3B).

This toolkit is not only suitable for an RNAi study but also for overexpression of protein by cloning of cDNA but not shRNAmir into the cloning sites between the 5′ LTR and the PGK promoter in the vectors. Furthermore, the multicolor feature of this toolkit can solve many experimental problems arising from fluorescent color incompatibility, such as lack of an appropriate fluorescent reporter in an RNAi/overexpression experiment in which fluorophore‐conjugated antibody or reagent must be used or the targeting cells express inherent fluorescent protein.

## Materials and Methods

### Reagents and Tools table

**Table:** 

Reagents/source	Reference or source	Identifier or catalog number
**Experimental models**		
293T	ATCC	CRL‐3216
U87	PUMC	N/A
U251	PUMC	N/A
A549	ATCC	CCL‐185
WM115	Dr. Zunling Li Lab	N/A
MC38	PUMC	N/A
NIH‐3T3	Dr. Zunling Li Lab	N/A
HT29	Dr. Yancun Yin	N/A
U937	Dr. Yancun Yin	N/A
MM.1R	Dr. Zunling Li Lab	N/A
EL4	Dr. Zunling Li Lab	N/A
P14 TCR transgenic mice	Dr. Lilin Ye Lab	N/A
C57BL/6N mice	Charles River/Vitalriver	213
LCMV Armstrong	Dr. Lilin Ye Lab	N/A
**Recombinant DNA**		
PLVX‐Puro	Clontech	632164
MIGR1	Addgene	27490
pMD2.G	Addgene	12259
PSPAX2	Addgene	12260
pCL‐Eco	Addgene	12371
Additional plasmids and more information	This study	Table 1
**Antibodies**		
Anti‐HDAC1	Proteintech	10197‐1‐AP
Anti‐BRG1	Proteintech	21634‐1‐AP
Anti‐MTA1	BETHYL	A300‐911A
Anti‐MTA2	Proteintech	17554‐1‐AP
Anti‐beta Actin	Proteintech	66009‐1‐Ig
Anti‐T‐bet	Biolegend	644801
Anti‐Pten	Proteintech	22034‐1‐AP
HRP‐conjugated Goat anti‐mouse IgG (H + L)	Proteintech	SA00001‐1
HRP‐conjugated Goat anti‐rabbit IgG (H + L)	Proteintech	SA00001‐2
APC/Fire™ 750 anti‐mouse CD4	Biolegend	100567
BV510™ anti‐mouse CD8a	Biolegend	100751
BV421™ anti‐rat CD90/mouse CD90.1	Biolegend	202529
PE anti‐mouse CD127 (IL‐7Rα)	Biolegend	135010
APC anti‐mouse/human KLRG1 (MAFA)	Biolegend	138412
PE/Cyanine7 anti‐rat CD90/mouse CD90.1	Biolegend	202518
APC anti‐mouse/human CD44 Antibody	Biolegend	103011
PerCP/Cyanine5.5 anti‐mouse CD8a	Biolegend	100733
APC anti‐mouse CD95 (Fas)	Biolegend	152603
APC anti‐human CD95 (Fas)	Biolegend	305611
**Oligonucleotides**		
miR‐AB or miR‐E based oligos	This study	Table 2
**Chemicals, enzymes and other reagents**		
Phusion High‐Fidelity DNA Polymerase	ThermoFisher	F530L
dNTP	Promega	U1515
DMEM high glucose	Hyclone	SH30022.1
Fetal bovine serum (FBS)	Hyclone	SV30208.02
PEI MAX	Polyscience	24765
ApaI restriction enzyme	NEB	R0114V
BamHI‐HF restriction enzyme	NEB	R3136V
EcoRI‐HF restriction enzyme	NEB	R3101V
XhoI restriction enzyme	NEB	R0146V
AfeI restriction enzyme	NEB	R0652S
ClaI restriction enzyme	NEB	R0197V
KpnI‐HF restriction enzyme	NEB	R3142V
PacI restriction enzyme	NEB	R0547S
PmeI restriction enzyme	NEB	R0560V
T4 DNA ligase	NEB	M0202S
100 bp ladder	NEB	N3231V
MojoSort™ Mouse CD8 T Cell Isolation Kit	Biolegend	480035
Xl‐10 Gold competent cells	Homemade	N/A
Nitrocellulose Membrane, 0.2 µm	Bio‐Rad	1620112
TEMED	Sigma	T9281‐25ML
Super Enhancer ECL Kit (For HRP)	Novland BioPharma	PWB‐001
TIANprep Mini Plasmid Kit	TIANGEN	DP103‐03
TIANquick Mini Purification Kit	TIANGEN	DP203‐02
TIANgel Mini Purification Kit	TIANGEN	DP208‐02
**Software**		
BD FACSDiva™	BD	N/A
GraphPad Prism 7.0	GraphPad	N/A
Flowjo V10	Flowjo	N/A
Image Lab 6.0.1	Bio‐Rad	N/A
miR‐AB Oligos Tool	This study	N/A

### Methods and Protocols

#### Cells and mice

293T, U87, U251, A549, WM115, MC38, NIH‐3T3, and HT29 cells were cultured in DMEM media supplemented with 10% fetal bovine serum (Hyclone, Logan, UT). U937, MM.1R, and EL4 cells were cultured in RPMI 1640 media supplemented with 10% fetal bovine serum (Hyclone, Logan, UT). P14 TCR transgenic mice were kindly provided by Dr. Lilin Ye (Institute of Immunology, Third Military Medical University, Chongqing, China). All mice were housed in our SPF facility. The Binzhou Medical University Animal Care and Use Committee approved all mouse experiments included in this work.

#### miR‐AB viral vectors design

Lentiviral vector pLVX‐Puro (Clontech) and retroviral vector MIGR1 (Plasmid #27490, Addgene) were used to create miR‐AB‐based viral vectors. The DNA sequence consisting of miR‐AB shRNAmir cassette, followed by PGK promoter, PacI, and PmeI restriction sites flanked Venus fluorescent protein‐coding sequence, and Woodchuck hepatitis virus post‐transcriptional regulation element (WPRE) was synthesized and cloned into pLVX‐Puro by AfeI and KpnI sites or into MIGR1 by BglII and ClaI sites. To create lentiviral vectors with miR‐AB cassette driven by multiple eukaryotic promoters, mouse CMV promoter, human EF1a promoter, CBh composite promoter, EH (EF1a/HTLV) composite promoter, and AH (hACTB/HTLV) composite promoter were synthesized with some modifications to remove restriction sites incompatible with this system while preserving the universal transcription factor‐binding sites. These modified promoters were cloned into the miR‐AB lentiviral vector by ClaI and AfeI sites to replace the endogenous human CMV promoter. To make lentiviral or retroviral miR‐AB vectors with multiple fluorescent protein reporters, eight fluorescent protein‐coding sequences were introduced by PacI and PmeI sites to replace endogenous Venus‐coding sequence. All these fluorescent protein‐coding sequences were codon optimized to remove restriction sites that conflict with the vectors. To facilitate the verification of miR‐AB vectors’ digestion by BamHI and ApaI, a ~800 bp stuffer sequence was cloned between BamHI and ApaI sites in all the above vectors for observation on agarose gel. All plasmids will be deposited at Addgene for ease of distribution.

#### miR‐AB Oligos Tool

This Microsoft Excel‐based tool is exclusively designed for converting of a 97 bp SplashRNA‐ or shERWOOD‐designed shRNAmir sequence to two short oligos for synthesis with desalt purification only. Users only need to copy and paste the 97‐bp sequence where indicated to generate two short oligos whose sequences are shown as red font on the right. If a ≠97 bp sequence is erroneously inputted, an alert will be prompted to user. To avoid error operation, the sheet is protected without encryption. This tool can convert up to 1,000 sequences. If more than 1,000 sequences are to be converted, the sheet is unprotected and the following steps are carried out: select one of the 1,000 formulated rows, drag the plus sign on the lower‐left corner down to where you want, and protect the sheet again. Two human FAS miR‐AB oligos have been created in this tool as a demonstration.

#### Cloning of SplashRNA or shERWOOD‐designed shRNAmirs into miR‐AB viral vectors

Target gene‐specific shRNAmir were designed by SplashRNA (http://splashrna.mskcc.org/) or shERWOOD (http://sherwood.cshl.edu:8080/sherwood/) online. The generated 97‐bp shRNAmir sequence was converted to two short oligos (75 bp and 67 bp) by miR‐AB Oligos Tool. The two oligos were synthesized (desalt purification only) at Genscript and resuspended in deionized water to 100 µM. To anneal the two oligos to generate double‐strand DNA with BamHI/ApaI overhangs, 1 µl of 75 bp oligo (100 µM), 1 µl of 67 bp oligo (100 µM), 10 µl of 10× annealing buffer (100 mM Tris‐HCl with pH 7.4, 10 mM EDTA and 0.5 M NaCl), and 88 µl of deionized water were mixed in a 1.5‐ml microcentrifuge tube, incubated at 95°C for 5 min on dry bath and slowly cooled to 50°C by shutting down the dry bath. The final concentration of the annealed oligo was 1 µM. Around 1–1.5 µg of miR‐AB vector was digested in 50 µl reaction volume with 1 µl of ApaI (NEB, Catalog No. R0114S), 1 µl of BamHI‐HF (NEB, Catalog No. R3136S), and 5 µl of 10× CutSmart Buffer for 30 min (longer incubation will decrease the cloning efficiency), purified by gel recovery and adjusted to 10 ng/µl with deionized water. The cut vectors (10 ng/µl) and the annealed oligos (1 µM) were used as stock and stored in humid container at 4°C. To make a ligation, the cut miR‐AB vectors was diluted to 0.5 ng/µl and the annealed oligos to 0.5 nM. 1 µl of vector, 1 µl of oligo, 0.25 µl of T4 DNA ligase (NEB, Catalog No. M0202S), 0.5 µl of 10× T4 DNA Ligase Reaction Buffer (NEB, Catalog No. B0202S), 0.5 µl of 50% PEG 4000, and 1.75 µl of deionized water (5 µl in total) were mixed and incubated at room temperature for 15 min. One microliter of the ligation product was used to transform XL10‐Gold or Stbl3 competent cells.

#### Lentivirus or retrovirus production

Healthy 293T cells were maintained in complete media (DMEM supplemented with 10% fetal bovine serum). One day before transfection, cells were plated in six‐well plates to reach > 80% confluence at transfection. One microgram of miR‐AB lentiviral vector together with packing plasmids (0.5 µg of pMD2.G and 1 µg of psPAX2) or 1 µg of miR‐AB retroviral vector together with 1 µg of pCL‐Eco packaging plasmid was used to transfect cells by PEI (polyethylenimine). The transfection media was removed 5 h after transfection, and 2 ml of fresh complete media was added. The viral supernatant was collected every 24 h. The first and second lentiviral collections, or the second and third retroviral collections were pooled, 0.45 µm filtered, and stored at −80°C.

#### Immunoblot analysis of RNAi efficiency *in vitro*

Human cell lines were cultured in appropriate culture media. Cells were plated in 24‐well plates 1 day before infection of lentivirus with Venus fluorescent protein as reporter. Forty‐eight after infection, fresh media was added for cell expansion and survival. Around 4–5 days after infection, cells were collected and lysed by boiling in 1× Laemmli sample buffer for 8 min. The lysates of equivalent number of cells were loaded onto SDS–PAGE for separation. After transfer to nitrocellulose membrane, target proteins were detected by appropriately diluted primary antibodies and then by HRP‐conjugated secondary antibody. The chemiluminescent signals were developed using Super Enhancer ECL Kit (Shanghai Novland, China), acquired by ChemiDoc XRS+ System, and analyzed by Bio‐Rad Image Lab software.

#### CD8^+^ T cells in vivo differentiation after Tbx21 RNAi

Naïve CD8^+^ T cells were isolated from P14 TCR transgenic mice that carried homozygous or heterozygous Thy1.1 allele using MojoSort™ Mouse CD8 Naïve T Cell Isolation Kit (Biolegend, Catalog No. 480043). 2 × 10^5^ cells were plated in 96‐well plate which was coated with 30 µg/ml of Goat anti‐Hamster IgG (H + L) Secondary Antibody (ThermoFisher Scientific, Catalog No. 31115), and activated by 1 µg/ml of Ultra‐LEAF™ Purified anti‐mouse CD3ε (Biolegend, Catalog No. 100340) and 1 µg/ml of Ultra‐LEAF™ Purified anti‐mouse CD28 ((Biolegend, Catalog No. 102116) for 16 h. Thy1.1^+/+^ and Thy1.1^+/−^ cells were transduced with miR‐AB or miR‐E‐based retrovirus, targeting *Tbx21* or *CD4* (control), respectively, for 4 h. 2.5 × 10^3^ of the transduced Thy1.1^+/+^ cells and 2.5 × 10^3^ of the transduced Thy1.1^+/−^ cells were mixed and adoptively transferred to naïve C57BL/6N mice (Thy1.2^+/+^) by retroorbital injection. The mice were then infected with 2 × 10^5^ PFU of LCMV‐Arm by intraperitoneal injection. Eight days after infection, mice were sacrificed by cervical dislocation and the spleens were removed for isolation of splenocytes by grinding on 70 µM cell strainer. After removal of red blood cells, the isolated splenocytes were stained with appropriately diluted antibodies including APC/Fire™ 750 anti‐mouse CD4 Antibody (Biolegend, Catalog No. 116019), PE/Cyanine7 anti‐mouse CD8a Antibody (Biolegend, Catalog No. 100721), Brilliant Violet 421™ anti‐rat CD90/mouse CD90.1 Antibody (Biolegend, Catalog No. 202529), PerCP/Cyanine5.5 anti‐mouse CD90.2 Antibody (Biolegend, Catalog No. 140321), PE anti‐mouse CD127 (IL‐7Rα) Antibody (Biolegend, Catalog No. 135010), and APC anti‐mouse/human KLRG1 (MAFA) Antibody (Biolegend, Catalog No. 138412) in PBS with 2% FBS for 20 min. After washing twice with PBS, the stained cells were acquired on flow cytometer (BD FACSCanto™). The FACS data were analyzed on FlowJo software.

#### FAS surface staining

3 × 10^3^ human or mouse cells were plated in 96‐well plates 1 day before infection with miR‐AB lentivirus with Venus fluorescent protein as reporter. Five days after infection, cells were washed with PBS two times and stained with APC‐conjugated anti‐human FAS Antibody (Biolegend, Catalog No. 305611) or APC‐conjugated anti‐mouse FAS Antibody (Biolegend, Catalog No. 152603) in PBS with 2% FBS for 20 min. After washing twice with PBS, cells were analyzed on flow cytometer (Beckman CytoFLEX). Live Venus‐positive cells were gated for analysis of FAS‐APC.

#### Multicolor‐barcoded RNAi

Two top‐ranked SplashRNA‐designed shRNAmirs targeting mouse CD127, CD90, CD44, or CD8 were cloned into miR‐AB retroviral vectors with Azurite, GFP, Ametrine, or mCherry2 as reporters, respectively. A SplashRNA‐designed shRNAmir‐targeting mouse CD4 or CD19 was cloned into each miR‐AB retroviral vectors with Azurite, GFP, Ametrine, or mCherry2 as reporters, which were used as RNAi control. Their packaged viruses were mixed at equal volume ration and used for transduction of activated mouse CD8^+^ T cells as described above. 72 h after transduction, cells were stained with fluorophore‐conjugated antibodies. Cells were acquired on BD FACSAria™ III Cell Sorter and analyzed by Flowjo software.

#### Sequences of shRNAmirs used in this study

All shRNAmirs information are described in Table 2.

## Author contributions

**Dapeng Wang:** Conceptualization; Resources; Data curation; Software; Formal analysis; Supervision; Funding acquisition; Investigation; Methodology; Writing—original draft; Project administration; Writing—review & editing. **Jianbo Xiu:** Validation. **Jiangyue Zhao:** Validation. **Junli Luo:** Supervision.

In addition to the CRediT author contributions listed above, the contributions in detail are:

DW designed this project, did all the experiments, and wrote the manuscript. JL guided this project. JX and JZ verified miR‐AB RNAi efficiency.

## Supporting information



Expanded View Figures PDF

Source Data for Figure 2

miR‐AB_Oligos_Tool

## Data Availability

No primary datasets have been generated and deposited.
