# Supplementary material for: miR‐AB, a miRNA‐based shRNA viral toolkit for multicolor‐barcoded multiplex RNAi at a single‐cell level
Source: EMBO Rep. 2022 Feb 24;23(4):e53691. doi: 10.15252/embr.202153691 (PMC8982575; doi:10.15252/embr.202153691)
Supplement: Supplementary file 1 — Expanded View Figures PDF [file EMBR-23-e53691-s001.pdf]

Expanded View Figures

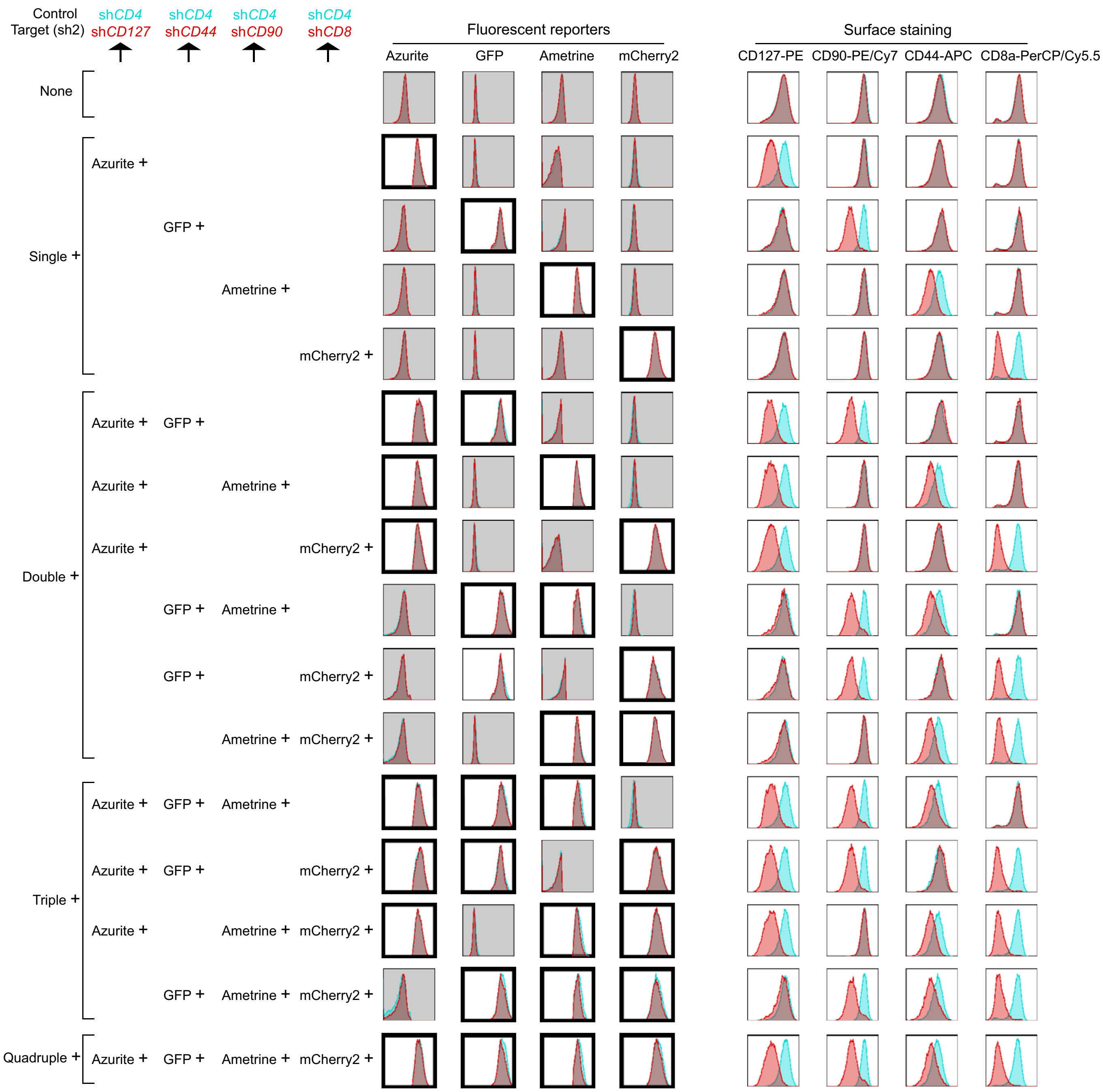

Figure EV1. Multicolor-barcoded multiplex RNAi by miR-AB retrovirus in CD8^{+} T cells.

This RNAi experiment was carried out as described in Fig 5 but used another SplashRNA-designed shRNAmirs targeting CD127, CD90, CD44, or CD8. CD4-specific shRNAmirs were used as controls. The data are representative of two biologically independent experiments.

© 2022 The Authors

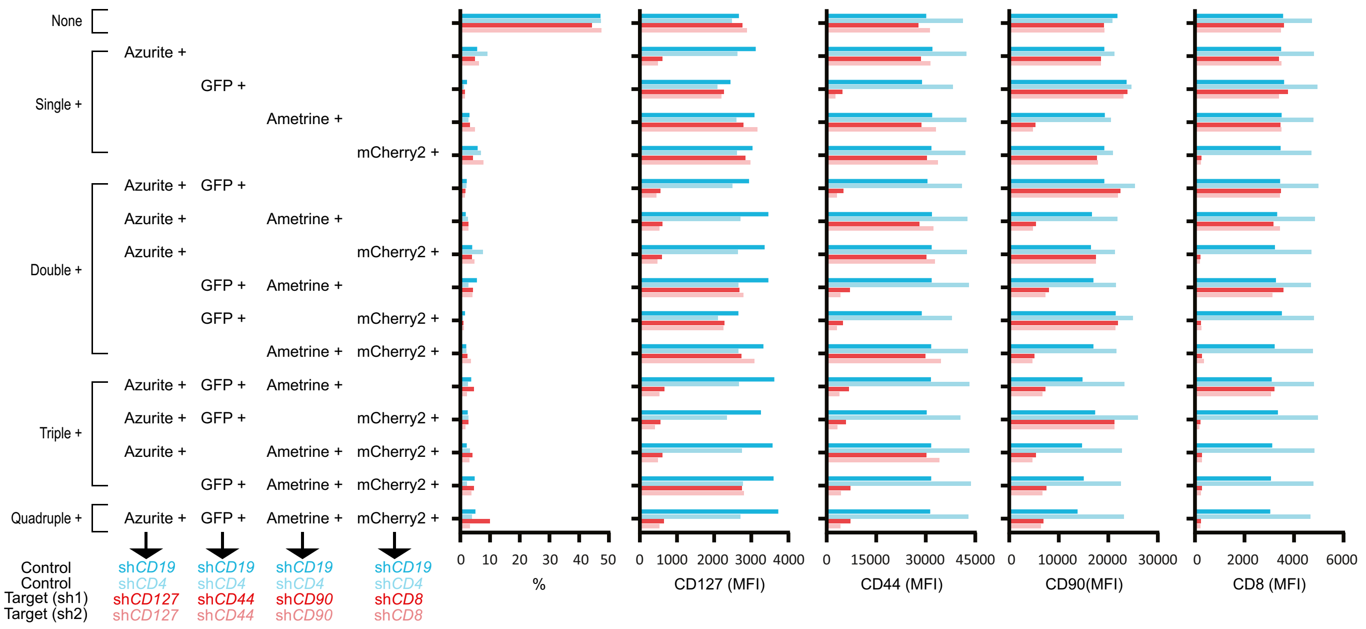

Figure EV2. RNAi efficiency quantification of FACS plots in Figs 5 and EV1.

Target gene expression was quantified by calculating its MFI in FACS plots. The percentage of all fluorescence-positive cells (untransduced, single, double, triple, and quadruple) were also shown (first column panels).

Figure EV3. Guidance of setting up a multicolor-barcoded multiplex RNAi.

A Multicolor-barcoded multiplex RNAi setup guidance. The fluorescent reporters for the RNAi assay and the fluorophores for RNAi verification or phenotypic marker staining are dependent on the lasers on a flow cytometer or fluorescent microscope. The optimal laser wavelength is indicated for each fluorescent reporter or fluorophore in a multicolor assay.

B A flowchart of performing a multicolor-barcoded RNAi experiment. Some suggestions and recommendations are listed below.

▶

© 2022 The Authors

A

Flow cytometer or Fluorescent microscope	2 lasers (488/633)	3 lasers (405/488/633)	4 lasers (405/488/561/633)
Multicolor assay (maximal)	3-colors	5-colors	6-colors
Fluorescent reporters	EGFP/Venus (488)	Azurite/mTagBFP2 (405)	Azurite/mTagBFP2 (405)
	mOrange (488)	Ametrine (405)	Ametrine (405)
	E2-Crimson (633)	EGFP/Venus (488)	EGFP/Venus (488)
		mOrange (488)	mOrange (561)
		E2-Crimson (633)	mCherry2 (561)
			E2-Crimson (633)
Fluorophores for RNAi verification or phenotypic marker staining	PerCP/Cy5.5 (488)	BV605 (405)	BV605 (405)
	PE/Cy7 (488)	BV711 (405)	BV711 (405)
	Alexa Fluor 700 (633)	BV785 (405)	BV785 (405)
	APC/Cy7 (633)	PerCP/Cy5.5 (488)	PerCP/Cy5.5 (488)
		PE/Cy7 (488)	PE/Cy7 (561)
		Alexa Fluor 700 (633)	Alexa Fluor 700 (633)
		APC/Cy7 (633)	APC/Cy7 (633)

B

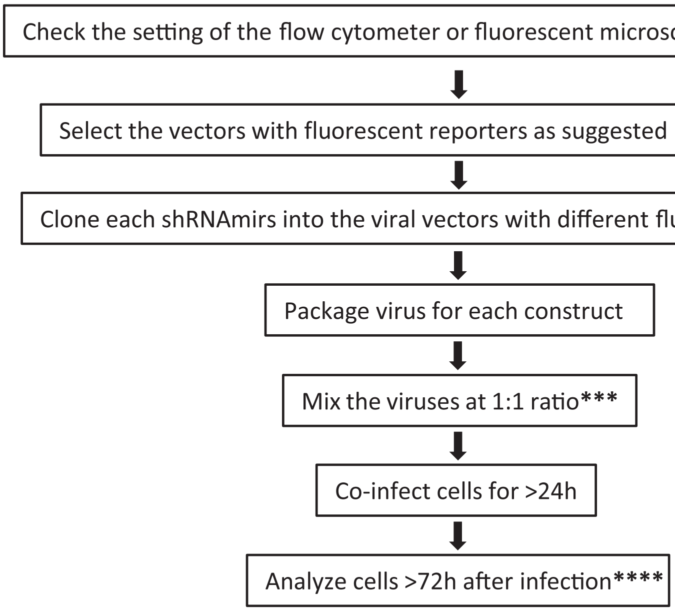

***** If you are not quite sure how to choose the fluorescent reports, go to flow core to request help. **Don't use Azurite and mTagBFP2 together, or EGFP and Venus together unless an advanced flow cytometer or microscope is available. Any other combinations among these eight fluorescent reporters can be used in most commercial flow cytometers and fluorescent microscopes.**

****** It is highly recommended to test if the shRNAmir in the vector context works in your cells. If it doesn't work well, choose a lentiviral miR-AB vector with different promoter.

******* If one virus results in far more or far less expression of its fluorescent reporter (not very likely), decrease or increase its usage.

******** When phenotypic marker is tested, you **MUST** make sure the fluorophore-conjugated antibody or reagent can be optically distinguished from the fluorescent reporter as suggested in the above table.

Figure EV3.
