# Supplementary figures and images for: miR‐AB, a miRNA‐based shRNA viral toolkit for multicolor‐barcoded multiplex RNAi at a single‐cell level
Source: EMBO Rep. 2022 Feb 24;23(4):e53691. doi: 10.15252/embr.202153691 (PMC8982575; doi:10.15252/embr.202153691)

Figure 2

A

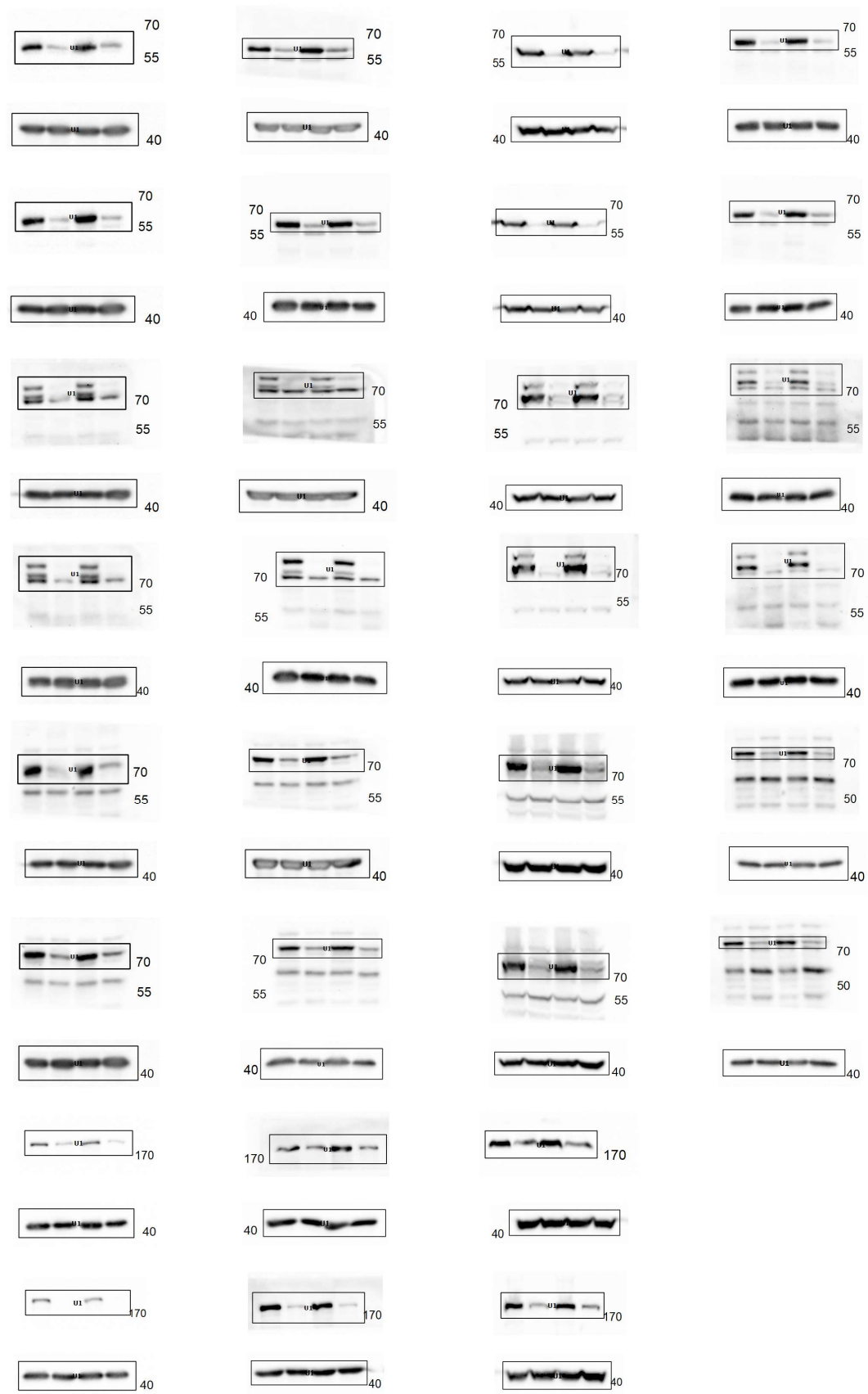

D

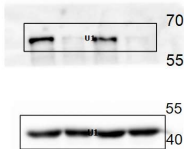

E

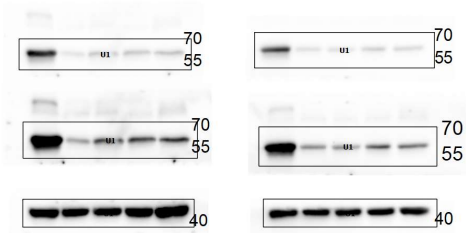

Supplement: Supplementary file 2 — Source Data for Figure 2 [file EMBR-23-e53691-s002.pdf]
