# Supplementary material for: miR‐AB, a miRNA‐based shRNA viral toolkit for multicolor‐barcoded multiplex RNAi at a single‐cell level
Source: EMBO Rep. 2022 Feb 24;23(4):e53691. doi: 10.15252/embr.202153691 (PMC8982575; doi:10.15252/embr.202153691)
Supplement: Supplementary file 4 [file EMBR-23-e53691-s004.pdf]

EMBO reports

# miR-AB, a miRNA-based shRNA viral toolkit for multicolor-barcoded multiplex RNAi at a single-cell level

Dapeng Wang, Jianbo Xiu, Jiangyue Zhao, and Jun-Li Luo
**DOI: 10.15252/embr.202153691**

*Corresponding author(s): Dapeng Wang (dwang@bzmc.edu.cn) , Jun-Li Luo (jlluo@scripps.edu)*



## Review Timeline:

| | |
|---|---|
| Submission Date: | 23rd Jul 21 |
| Editorial Decision: | 27th Sep 21 |
| Revision Received: | 13th Dec 21 |
| Editorial Decision: | 18th Jan 22 |
| Revision Received: | 21st Jan 22 |
| Accepted: | 30th Jan 22 |

*Editor: Martina Rembold*


## Transaction Report:

(Note: With the exception of the correction of typographical or spelling errors that could be a source of ambiguity, letters and reports are not edited. Depending on transfer agreements, referee reports obtained elsewhere may or may not be included in this compilation. Referee reports are anonymous unless the Referee chooses to sign their reports.)

Dear Dr. Wang

Thank you for the submission of your research manuscript to our journal. I am sorry for the delay in handling your manuscript, but we have only recently received the full set of referee reports that is copied below.

As you will see, both referees acknowledge that the multiplex reporter system will be a valuable resource for the community. However, referees 1 and 2 also point out several technical concerns and have a number of suggestions for how the study should be strengthened, and I think that all of them should be addressed. Please also provide further validation of the RNAi efficiency by at least analysing a subset of shRNAmiRs at single copy level as suggested by referee 2.

Given these constructive comments, we would like to invite you to revise your manuscript with the understanding that the referee concerns (as detailed above and in their reports) must be fully addressed and their suggestions taken on board. Please address all referee concerns in a complete point-by-point response. Acceptance of the manuscript will depend on a positive outcome of a second round of review. It is EMBO reports policy to allow a single round of revision only and acceptance or rejection of the manuscript will therefore depend on the completeness of your responses included in the next, final version of the manuscript.

Revised manuscripts should be submitted within three months of a request for revision; they will otherwise be treated as new submissions. Please contact us if a 3-months time frame is not sufficient for the revisions so that we can discuss the revisions further.

***IMPORTANT NOTE: we perform an initial quality control of all revised manuscripts before re-review. Your manuscript will FAIL this control and the handling will be DELAYED if the following APPLIES:

1) A data availability section is missing.
2) Your manuscript contains error bars based on n=2. Please use scatter blots showing the individual datapoints in these cases. The use of statistical tests needs to be justified.

When submitting your revised manuscript, please carefully review the instructions that follow below. Failure to include requested items will delay the evaluation of your revision.***

When submitting your revised manuscript, we will require:

1) a .docx formatted version of the manuscript text (including legends for main figures, EV figures and tables). Please make sure that the changes are highlighted to be clearly visible. Please also combine the Results and Discussion section since your study will be published in the 'Reports' format.

2) individual production quality figure files as .eps, .tif, .jpg (one file per figure).
Please download our Figure Preparation Guidelines (figure preparation pdf) from our Author Guidelines pages https://www.embopress.org/page/journal/14693178/authorguide for more info on how to prepare your figures.

3) a .docx formatted letter INCLUDING the reviewers' reports and your detailed point-by-point responses to their comments. As part of the EMBO Press transparent editorial process, the point-by-point response is part of the Review Process File (RPF), which will be published alongside your paper.

4) a complete author checklist, which you can download from our author guidelines (). Please insert information in the checklist that is also reflected in the manuscript. The completed author checklist will also be part of the RPF.

5) Please note that all corresponding authors are required to supply an ORCID ID for their name upon submission of a revised manuscript (). Please find instructions on how to link your ORCID ID to your account in our manuscript tracking system in our Author guidelines
()

6) We replaced Supplementary Information with Expanded View (EV) Figures and Tables that are collapsible/expandable online. A maximum of 5 EV Figures can be typeset. EV Figures should be cited as 'Figure EV1, Figure EV2" etc... in the text and their respective legends should be included in the main text after the legends of regular figures.

- For the figures that you do NOT wish to display as Expanded View figures, they should be bundled together with their legends in a single PDF file called *Appendix*, which should start with a short Table of Content. Appendix figures should be referred to in the main text as: "Appendix Figure S1, Appendix Figure S2" etc. See detailed instructions regarding expanded view here:

- Additional Tables/Datasets should be labeled and referred to as Table EV1, Dataset EV1, etc. Legends have to be provided in a separate tab in case of .xls files. Alternatively, the legend can be supplied as a separate text file (README) and zipped together with the Table/Dataset file.

7) Please note that a Data Availability section at the end of Materials and Methods is now mandatory. In case you have no data that requires deposition in a public database, please state so instead of refereeing to the database.
See also < https://www.embopress.org/page/journal/14693178/authorguide#dataavailability>). Please note that the Data Availability Section is restricted to new primary data that are part of this study.

8) Figure legends and data quantification:

The following points must be specified in each figure legend:
- the name of the statistical test used to generate error bars and P values,
- the number (n) of independent experiments (please specify technical or biological replicates) underlying each data point,
- the nature of the bars and error bars (s.d., s.e.m.)
- If the data are obtained from n {less than or equal to} 2, use scatter blots showing the individual data points.

Discussion of statistical methodology can be reported in the materials and methods section, but figure legends should contain a basic description of n, P and the test applied.
See also the guidelines for figure legend preparation:
https://www.embopress.org/page/journal/14693178/authorguide#figureformat

- Please also include scale bars in all microscopy images.

9) We would kindly ask you to use 'Structured Methods', our new Materials and Methods format, which is mandatory for Method papers (see example: ). The Materials and Methods section should include a Reagents and Tools Table (listing key reagents, experimental models, software and relevant equipment and including their sources and relevant identifiers) followed by a Methods and Protocols section in which methods can be described using a step-by-step protocol format with bullet points. More information is available at .

10) We would also encourage you to include the source data for figure panels that show essential data. Numerical data should be provided as individual .xls or .csv files (including a tab describing the data). For blots or microscopy, uncropped images should be submitted (using a zip archive if multiple images need to be supplied for one panel). Additional information on source data and instruction on how to label the files are available .

11) Our journal encourages inclusion of *data citations in the reference list* to directly cite datasets that were re-used and obtained from public databases. Data citations in the article text are distinct from normal bibliographical citations and should directly link to the database records from which the data can be accessed. In the main text, data citations are formatted as follows: "Data ref: Smith et al, 2001" or "Data ref: NCBI Sequence Read Archive PRJNA342805, 2017". In the Reference list, data citations must be labeled with "[DATASET]". A data reference must provide the database name, accession number/identifiers and a resolvable link to the landing page from which the data can be accessed at the end of the reference. Further instructions are available at .

12) As part of the EMBO publication's Transparent Editorial Process, EMBO reports publishes online a Review Process File to accompany accepted manuscripts. This File will be published in conjunction with your paper and will include the referee reports, your point-by-point response and all pertinent correspondence relating to the manuscript.

You are able to opt out of this by letting the editorial office know (emboreports@embo.org). If you do opt out, the Review Process File link will point to the following statement: "No Review Process File is available with this article, as the authors have chosen not to make the review process public in this case."

We would also welcome the submission of cover suggestions, or motifs to be used by our Graphics Illustrator in designing a cover.

I look forward to seeing a revised version of your manuscript when it is ready. Please let me know if you have questions or comments regarding the revision.

You can use this link to submit your revision: https://embor.msubmit.net/cgi-bin/main.plex

Yours sincerely,

Martina Rembold, PhD

Senior Editor
EMBO reports

**********************

Referee #1:

The authors describe in this manuscript a set of tools to facilitate the combinatorial gene knockdowns using microRNA-based shRNA. The main advances are a simplification in the shRNA cloning strategy and an array of fluorescent proteins added to the shmiRNA expression vector to trace combinatorial perturbations. First, the ease of cloning into the novel shmiRNA vector (miR-AB) is highlighted, while also showing the efficiency of knockdowns using said vector. Several versions of that vector (different promoters and fluorescent proteins) are then validated by showing knockdowns of a small set of genes. This panel of vectors will be a valuable resource for combinatorial knockdowns. Lastly, combinatorial knockdowns are then performed up to a maximum of four perturbations per cell. The performed experiments and presented data are technically appropriate and scientifically sound and of value to the scientific community. However, some experiments and in particular some figures would greatly benefit from some clarification to make the manuscript better understandable and underline the key messages.
1) Figure 3 and figure 5 should be clarified. First, quantifying and visualizing the level of successful knockdown will greatly improve the message of these two experiments. To do so, one could add a gating line based on the expression level in the parental cell lines - in particular for the promoter comparison. Figure 5 needs the knockdown quantification for two reasons. On one hand, having a table to summarize these levels will make the figure easier to understand. But more importantly, the table should also include the percentages of double/triple and quadruple knockdowns. This data is very important and can only be inferred from the histograms.

Minor points:

1) A baseline such as a non-stained or even better an isotype stained control should be added to cell surface marker stained flow cytometry data as a baseline representing non-expressing cells.

2) It would be nice to mention the time and cost saving of miR-AB vs miR-E in the discussion.

3) This statement in the discussion should be toned down: 'Since all miR-AB-based shRNAmirs used in this study showed potent RNAi efficacy, miR-AB, in combination of SplashRNA or shERWOOD, would outcompete most of the current RNAi tools which always need a pilot experiment to validate their candidate shRNAs.'
This statement appears premature given the limited number of shmiRNA assessed here. A more comprehensive analysis (eg. targeting essential genes with multiple shmiRNA targeting each gene and confirmation of the efficiency of knockdown of this larger panel) would be needed to state that shmiRNA is superior to previously reported strategies.

4) The section in the discussion on fluorescent protein compatibility should be streamlined and clarified. Together with the informative tables with recommendations on panel design, this section can be kept much simpler and less confusing.

5) Several typos should be corrected. See for example:
Page3 - Dice instead of Dicer
Page 5 - packaging instead of packaged
Page 7- picogram instead of picograms
Figure 1B - Transform instead of transfect 293T cells
Throughout the manuscript, the authors sometime switch between first person (I) and third person (we).

Referee #2:

1. Summary
Wang et al. develop "miR-AB", a novel shRNAmiR-system based on the human miR30A backbone. In miR-AB, restriction sites are introduced into the lower stem region which facilitates cloning since annealed oligos can be used directly for cloning into the miR-AB backbone, thereby by omitting laborious and expensive PCR-amplification of oligos. The authors develop the system into a versatile toolkit and show that miR-AB works as polycistronic construct after different fluorescent proteins and can be driven by several, widely used eukaryotic promoters. The manuscript has merits, especially regarding the multicolor-barcording RNAi. However, I have some reservations about the novelty and technical execution of parts of the study that should be addressed before a potential publication.

2. Key results
miR-AB is a versatile and easy to use RNAi trigger in different contexts.

3. Clarity and context
The manuscript is clearly written and the goals are clear. The reader can follow the course of analysis and conclusions drawn from the data.

4. Major points

4.1 Results: "De novo cloning of shRNAmir into miR-AB is simple, inexpensive and error-proof"

In this part the authors describe the development of miR-AB. miR-AB was obtained by introducing ApaI/BamHI restriction sites into the lower stem and bulge of the widely used miR30-E backbone. By moving the restriction sites closer together, it is possible to clone directly two annealed oligos of 75 bp and 65 bp length into the backbone, whereas in the classical miR30-E system (Fellmann/Zuber 2013) the oligos needed are longer (97mers) and have to be amplified by PCR before cloning into the miR30-E backbone. However, this strategy to rapidly clone shRNAmiRs is not novel. Several groups, e.g. Adams et al. (An optimized lentiviral vector system for conditional RNAi and efficient cloning of microRNA embedded short hairpin RNA libraries Biomaterials 2017) have developed similar systems, albeit they mostly use BsmBI (Type IIS) restriction sites. The authors claim that these systems suffer from low cloning efficacy but do not provide data to support this claim. A head-to-head comparison between these systems and miR30-AB has not been performed. Given that complex shRNAmiR librarys have been cloned and validated by NGS with these systems, efficacy and accuracy does not seem to be a bottleneck there. At least, it should be mentioned that similar systems have been developed before and appropriately referenced. Claims about the superiority of miR30-AB should be deleted when there is no data to support them.

4.2 Results: "miR-AB shows outstanding RNAi efficiency in vitro"

In this paragraph, the knockdown efficacy is compared between the novel miR30-AB and the miR30-E shRNAmiR system for different shRNAs predicted by SplashRNA. However, I have great concerns about the validity of the results of this paragraph, since the authors have performed the knockdown experiments in cell lines where "A nearly 100% of transduction efficiency was achieved after 72h of infection". This means, the authors worked with cell lines had multiple, possibly tens or even hundreds of integrations of the viruses encoding the shRNAmiRs. Since maximal 100% of the cells can be green after transduction it remains unclear whether cells harboring a comparable number of integrations and therefore vector dosage and shRNAmiR payload were compared in these experiments. It is nowhere mentioned at which MOI the cells were transduced. The efficacy of RNAi triggers must generally be compared at singly copy level (Fellmann et al. Cell Reports 2013), this means a maximum of 30% of transduced cells is allowed. To rescue this experiment the authors could determine the vector copy number in the samples or at least state at which MOI the cells where transduced. However, at least three independent shRNAmiRs should be evaluated at single copy level (not necessarily in all cell lines) to support the conclusion that miR30-AB is non-inferior to miR30-E. SplashRNA predicts very powerful RNAi-triggers, which can be difficult to compare due to their high knockdown potency. E.g. the one of the most powerful shRNAs Pten.1523 performs well even when expressed from the original miR-30 backbone. Therefore I would suggest to include some medium-power shRNAmiRs, such as Pten.1524 or Pten.932 (mouse - use NIH3T3) to compare knockdown potency in RNAi triggers that have the potential to improve or deteriorate when expressed from different miR30 backbones.

4.3 Results: "Multiple eukaryotic promoters guarantee miR-AB-based RNAi in various cell types"
"These new constructs were packaging in 293T cells as effectively as the original vector with the human CMV promoter and maintained the intensity of the Venus reporter." I presume the authors wish to state that viral titers are the same for the constructs with different promoters. If this is the case data to support this notion should be shown. The FACS plots in Figure 3 should be supported by the MFIs in a supplementary table to support the conclusions drawn in this paragraph.

4.4 Results: "Construction of a multi-promoter and multicolor miR-AB-based viral toolkit"
"These fluorescent protein-carrying miR-AB viral vectors can produce high viral titers and generate" - the titers achieved with these constructs on average should be mentioned to support this notion.
Second, it would be important to include cell viability assays to show whether the expression of multiple shRNAs and fluorescence proteins is toxic. To determine even subtle effects I would suggest to transduce cells with the 4 different vectors (Azurite, EGFP, Ametrine, mCherry) each harboring a neutral shRNA and follow them over time in a bulk culture to see whether cells harboring more FPs are outdiluted slowly over time. It is important to show this stability over time since it can greatly impact in vivo experiments.

## Response to the reviewers

Referee #1:

*The authors describe in this manuscript a set of tools to facilitate the combinatorial gene knockdowns using microRNA-based shRNA. The main advances are a simplification in the shRNA cloning strategy and an array of fluorescent proteins added to the shmiRNA expression vector to trace combinatorial perturbations. First, the ease of cloning into the novel shmiRNA vector (miR-AB) is highlighted, while also showing the efficiency of knockdowns using said vector. Several versions of that vector (different promoters and fluorescent proteins) are then validated by showing knockdowns of a small set of genes. This panel of vectors will be a valuable resource for combinatorial knockdowns. Lastly, combinatorial knockdowns are then performed up to a maximum of four perturbations per cell. The performed experiments and presented data are technically appropriate and scientifically sound and of value to the scientific community. However, some experiments and in particular some figures would greatly benefit from some clarification to make the manuscript better understandable and underline the key messages.*

**Our response**:

We are very pleased that you are interested in the study and are thankful for your helpful comments. We revised the main text and Figures as you suggested. Moreover, we did new experiments to show miR-AB's knockdown efficiency at single copy level (Figure 2E, 2F) and multiple shRNAmir transduction is not cytotoxic (Figure 4F). Furthermore, we developed new vectors for easy test of the activity of the promoters used in this toolkit (Figure 4D). According to the format requirement of EMBO reports, the Results and Discussion were combined and the text relating to the new data or being dramatically revised were highlighted for your easy recognition.

*1) Figure 3 and figure 5 should be clarified. First, quantifying and visualizing the level of successful knockdown will greatly improve the message of these two experiments. To do so, one could add a gating line based on the expression level in the parental cell lines - in particular for the promoter comparison. Figure 5 needs the knockdown quantification for two reasons. On one hand, having a table to summarize these levels will make the figure easier to understand. But more importantly, the table should also include the percentages of double/triple and quadruple knockdowns. This data is very important and can only be inferred from the histograms.*

**Our response**:

As you suggested, Figure 3 was quantified by showing the MFI of APC-FAS (bottom panels). Figure 5 and Figure EV1 were also quantified by showing the MFI of target genes (Figure EV2). The percentages of all single, double, triple and quadruple knockdown cells were included in Figure EV2. "The overall percentage of these populations was >50% (untransduced cell percentage was <50%, top bars in Fig EV2), indicating these viruses had high viral titers because primary CD8+ T cells are harder to be infected than cell lines. The lowest individual percentage of these population was >1%, indicating there are enough cells for FACS analysis of each population in this

assay (hundreds of cells can give a clear FACS population).” have been included in the text.

As another reviewer suggested, we used MFI but not gating line to quantify the expression of target genes, because these cells are homogeneous, and all cell lines express FAS (Figure 3) and all activated CD8+ T cells express CD127, CD44, CD90 and CD8 (Figure 5 and EV1). A gating line is commonly used for separating positive population from negative population in a heterogeneous cell population. If we look at the histograms of the control shRNA and FAS shRNA in Figure 3, they both have only one peak. Moreover, the FAS shRNA peak locates on the left of control shRNA peak, indicating all cells express FAS. If some cells are FAS negative in the control shRNA peak, the peak of FAS shRNA should be narrower and not closer to the left than control shRNA peak.

*Minor points:*

*1) A baseline such as a non-stained or even better an isotype stained control should be added to cell surface marker stained flow cytometry data as a baseline representing non-expressing cells.*

**Our response**:

To show RNAi effects in our FACS experiments, we used the commonly used method in immunology, i.e. comparison between control shRNA-transduced cells and target gene specific shRNA-transduced cells after staining with same target protein-specific antibody. We didn’t use isotype control since it is hard to get the basal expression level from it. An ideal isotype control should guarantee same species, same heavy chain and light chain class, same fluorochrome and same fluochrome:antibody ratio. This is almost impossible. “Same” isotype controls from different company or different batches from same company always give different results. Therefore, some scientists argue, even abandon the use of isotype controls in some experiments. If we go to Purdue Cytometry List, the best cytometry forum, we will see lots of arguments relating to isotype controls. In my opinion, Isotype controls are most useful when a new fluochrome-conjugated antibody is developed and needs to be validated. In this study, we used reliable fluorophore conjugated monoclonal antibodies that have been wildly used for long time.

To test the knockdown efficiency of endogenous gene, the best way is to use target gene knockout cells as negative control. After staining of knockout cells and the cells to be analyzed with the same target gene-specific antibody, we can get accurate information of the target gene expression changes. However, knockout cells are not always available for some experiment.

The purpose of FACS experiments in this study is to show the differential promoter activity (Figure 3) or the feasibility of carrying out a multicolor-based multiplex RNAi (Figure 5 and EV1). The exact RNAi efficiency has been shown in the western blot data

and GFP RNAi FACS data in Figure 2. The GFP RNAi FACS data clearly showed both miR-AB and miR-E backbone mediated strong RNAi by Sh2 and Sh3 (very few low-MFI GFP+ cells left).

*2) It would be nice to mention the time and cost saving of miR-AB vs miR-E in the discussion.*

**Our response**:

The time and cost saving of miR-AB vs miR-E have been included in the text as follows: "This new approach significantly reduces the cost and time in production of shRNAmir. Specifically, cloning of a shRNAmir by miR-AB only costs the synthesis of 142bp oligos (75bp + 67bp), takes 0.5 hour for annealing of miR-AB oligos and requires minimal labor (mixing and pipetting the oligos), while its cloning by PCR is more cost-ineffective and labor-intensive, including the synthesis of 97bp oligo, running a PCR, separating PCR product on agarose gel, gel purification of PCR product and restriction enzyme digestion of PCR product followed by purification and quantification."

*3) This statement in the discussion should be toned down: 'Since all miR-AB-based shRNAmirs used in this study showed potent RNAi efficacy, miR-AB, in combination of SplashRNA or shERWOOD, would outcompete most of the current RNAi tools which always need a pilot experiment to validate their candidate shRNAs.*

*This statement appears premature given the limited number of shmiRNA assessed here. A more comprehensive analysis (eg. targeting essential genes with multiple shmiRNA targeting each gene and confirmation of the efficiency of knockdown of this larger panel) would be needed to state that shmiRNA is superior to previously reported strategies.*

**Our response**:

Your suggestion is pretty accurate because we didn't make a comprehensive comparison of shRNAmir vs shRNA. Therefore, this sentence has been removed.

*4) The section in the discussion on fluorescent protein compatibility should be streamlined and clarified. Together with the informative tables with recommendations on panel design, this section can be kept much simpler and less confusing.*

**Our response**:

As you suggested, the text was revised and Figure EV3 was made to clarify the compatibility of these fluorescent proteins. To put it in a simple way, we highlighted in Figure EV3 "Don't use Azurite and mTagBFP2 together, or GFP and Venus together unless an advanced flow cytometer or microscope is available. Any other combinations among these eight fluorescent reporters can be used in most commercial flow cytometers and fluorescent microscopes". In Figure EV3, a detailed protocol of setting

up a multicolor assay was included and some suggestions and recommendations were given for avoiding mistakes.

*5) Several typos should be corrected. See for example:*

*Page3 - Dice instead of Dicer*

*Page 5 - packaging instead of packaged*

*Page 7- picogram instead of picograms*

*Figure 1B - Transform instead of transfect 293T cells*

*Throughout the manuscript, the authors sometime switch between first person (I) and third person (we).*

**Our response**:

Thank you for your pointing out the mistakes. We really appreciate. All typos have been corrected.

Referee #2:

*1. Summary*

*Wang et al. develop "miR-AB", a novel shRNAmiR-system based on the human miR30A backbone. In miR-AB, restriction sites are introduced into the lower stem region which facilitates cloning since annealed oligos can be used directly for cloning into the miR-AB backbone, thereby by omitting laborious and expensive PCR-amplification of oligos. The authors develop the system into a versatile toolkit and show that miR-AB works as polycistronic construct after different fluorescent proteins and can be driven by several, widely used eukaryotic promoters. The manuscript has merits, especially regarding the multicolor-barcording RNAi. However, I have some reservations about the novelty and technical execution of parts of the study that should be addressed before a potential publication.*

**Our response:**

We are very thankful for your valuable comments and suggestions. We did new experiments and revised the text and data according to your and other reviewer's suggestions. Furthermore, we developed new vectors for easy test of the activity of the promoters used in this toolkit (Figure 4D). According to the format requirement of EMBO reports, the Results and Discussion were combined and the text relating to the new data or being dramatically revised were highlighted for your easy recognition.

*2. Key results*

*miR-AB is a versatile and easy to use RNAi trigger in different contexts.*

*3. Clarity and context*

*The manuscript is clearly written and the goals are clear. The reader can follow the course of analysis and conclusions drawn from the data.*

*4. Major points*

*4.1 Results: "De novo cloning of shRNAmir into miR-AB is simple, inexpensive and error-proof"*

*In this part the authors describe the development of miR-AB. miR-AB was obtained by introducing ApaI/BamHI restriction sites into the lower stem and bulge of the widely used miR30-E backbone. By moving the restriction sites closer together, it is possible to clone directly two annealed oligos of 75 bp and 65 bp length into the backbone, whereas in the classical miR30-E system (Fellmann/Zuber 2013) the oligos needed are longer (97mers) and have to be amplified by PCR before cloning into the miR30-E backbone. However, this strategy to rapidly clone shRNAmiRs is not novel. Several groups, e.g. Adams et al. (An optimized lentiviral vector system for conditional RNAi and efficient cloning of microRNA embedded short hairpin RNA libraries Biomaterials 2017) have developed similar systems, albeit they mostly use BsmBI (Type IIS) restriction sites. The authors claim that these systems suffer from low cloning efficacy but do not provide data to support this claim. A head-to-head comparison between these systems and miR30-AB has not been performed. Given that complex shRNAmiR librarys have been cloned and validated by NGS with these systems, efficacy and accuracy does not seem to be a bottleneck there. At least, it should be mentioned that similar systems have been developed before and appropriately referenced. Claims about the superiority of miR30-AB should be deleted when there is no data to support them.*

**Our response:**

As you suggested, the BsmBI-based paper was referenced. Since we didn’t make a head-to-head comparison between miR-AB and BsmBI-based backbone, we don’t know if their cloning efficacy and reliability are similar or different. Our miR-AB based shRNAmir cloning doesn’t need oligo phosphorylation, which is an advantage over BsmBI-based backbone. Moreover, BsmBI (3.7$/ul, NEB) is more expensive than BamHI (0.144$/ul, NEB) and ApaI (0.62$/ul, NEB). These words “simple, inexpensive and error-proof” are only a description of our data in Figure 1, not a claim that miR-AB is superior to any other approaches. Any other words or phrases that might remind audience of miR-AB’s superiority have been removed. The aim of these study is to develop a RNAi toolkit that can be used to set up a multicolor-based multiplex RNAi assay. These miR-AB vectors can provide a choice for RNAi users.

*4.2 Results: "miR-AB shows outstanding RNAi efficiency in vitro"*

*In this paragraph, the knockdown efficacy is compared between the novel miR30-AB and the miR30-E shRNAmiR system for different shRNAs predicted by SplashRNA. However, I have great concerns about the validity of the results of this paragraph, since the authors have performed the knockdown experiments in cell lines where "A nearly 100% of transduction efficiency was achieved after 72h of infection". This means, the authors worked with cell lines had multiple, possibly tens or even hundreds of integrations of the viruses encoding the shRNAmiRs. Since maximal 100% of the cells can be green after transduction it remains unclear whether cells harboring a comparable number of integrations and therefore vector dosage and shRNAmiR payload were compared in these experiments. It is nowhere mentioned at which MOI the cells were transduced. The efficacy of RNAi triggers must generally be compared at singly copy level (Fellmann et al. Cell Reports 2013), this means a maximum of 30% of transduced cells is allowed. To rescue this experiment the authors could determine the vector copy number in the samples or at least state at which MOI the cells where transduced. However, at least three independent shRNAmiRs should be evaluated at single copy level (not necessarily in all cell lines) to support the conclusion that miR30-AB is non-inferior to miR30-E. SplashRNA predicts very powerful RNAi-triggers, which can be difficult to compare due to their high knockdown potency. E.g. the one of the most powerful shRNAs Pten.1523 performs well even when expressed from the original miR-30 backbone. Therefore I would suggest to include some medium-power shRNAmiRs, such as Pten.1524 or Pten.932 (mouse - use NIH3T3) to compare knockdown potency in RNAi triggers that have the potential to improve or deteriorate when expressed from different miR30 backbones.*

**Our response**:

Your comments and suggestions are pretty instructive and meaningful. As you suggested, we tested Pten.1524 and Pten.932 RNAi efficiency in the context of less than 30% transduction efficiency. After FACS sorting of GFP+ 3T3 cells, we did western blot to determine the expression level of Pten (Figure 2E). Consistent with the miR-E paper, both Pten.1524 and Pten.932 resulted in a significant reduction of Pten protein level at low transduction efficiency. These potent shRNAmirs displayed no significant knockdown difference in the context of miR-AB vs. miR-E.

To more intuitively show miR-AB’s RNAi efficiency at single copy level, we carried out a flow cytometry-based experiment to show transduction efficiency and RNAi efficiency simultaneously. To this end, we cloned three shRNAmirs targeting the GFP reporter in a lentiviral vector with miR-AB or miR-E backbone and analysed their RNAi potency after transduction of MC38 or 293T cells. AS shown in Fig 2F, in the context of less than 30% transduction efficiency, these shRNAmirs showed potent (Sh1 and Sh2) or moderate (Sh3) knockdown efficiency (indicated by *) in GFP+ cells. Their RNAi potency was comparable between in the miR-AB vs. miR-E backbone (indicated by NA). It should be noted that the medium-power Sh3 data is more meaningful to compare miR-AB vs. miR-E than Sh1 and Sh2, because GFP was almost undetectable in Sh1 and Sh2 transduced cells (their MFI only represents the leftover GFP+ cells after RNAi). This paragraph has been included in the text.

4.3 Results: "Multiple eukaryotic promoters guarantee miR-AB-based RNAi in various cell types"

"These new constructs were packaging in 293T cells as effectively as the original vector with the human CMV promoter and maintained the intensity of the Venus reporter." I presume the authors wish to state that viral titers are the same for the constructs with different promoters. If this is the case data to support this notion should be shown. The FACS plots in Figure 3 should be supported by the MFIs in a supplementary table to support the conclusions drawn in this paragraph.

**Our response:**

The titers of different promoter-based lentivirus were shown in the text as follows "A cost-effective transfection of 293T cells with these constructs by PEI (~40% transfection efficiency) can generate 3-5 × $10^6$ transduction unit titers of virus from one well of six-well plate. Commercial transfection reagent can generate higher titers by increasing transfection efficiency." As you and another reviewer suggested, Figure 3, Figure 5 and Figure EV1 were quantified by showing the MFI of target proteins.

*4.4 Results: "Construction of a multi-promoter and multicolor miR-AB-based viral toolkit"*

*"These fluorescent protein-carrying miR-AB viral vectors can produce high viral titers and generate" - the titers achieved with these constructs on average should be mentioned to support this notion.*

*Second, it would be important to include cell viability assays to show whether the expression of multiple shRNAs and fluorescence proteins is toxic. To determine even subtle effects I would suggest to transduce cells with the 4 different vectors (Azurite, EGFP, Ametrine, mCherry) each harboring a neutral shRNA and follow them over time in a bulk culture to see whether cells harboring more FPs are outdiluted slowly over time. It is important to show this stability over time since it can greatly impact in vivo experiments.*

**Our response**:

In this study, we mainly used retrovirus carrying different fluorescent proteins to infect primary mouse CD8+ T cells (Figure 4, 5 and EV1), which are harder to be infected than cell lines. Immunologists always use undiluted retrovirus to infect primary CD8+ T cells for short time (4h in this study) because even though highly-concentrated virus was used, the transduction efficiency would not be higher than 80-90%. As shown in our multicolor assay using multiple retrovirus carrying different fluorescent reporters at 1:1 ratio (Figure S4), more than 50% of cells were infected by unconcentrated virus (untransduced cell percentage is <50%), indicating these retroviral vectors can produce high viral titers. Moreover, each FACS plot in Figure 4E clearly showed two fluorescent reporter single positive cell populations (top left quadrant and bottom right quadrant) have similar percentage, indicating all the viral vectors were packaged efficiently. Our recent retrovirus titering assay using CD8+ T cells showed one well of 6-well plate by PEI transfection produced ~0.5-1 ×$10^6$ transfection unit of virus. To clarify, "The overall percentage of these populations was >50% (untransduced cell percentage was <50%, top bars in Fig EV2), indicating these viruses had high viral titers because primary CD8+ T cells are harder to be infected than cell lines. The lowest individual percentage of

these population was >1%, indicating there are enough cells for FACS analysis of each population in this assay (hundreds of cells can give a clear FACS population).” was added in the main text.

As you suggested, we transduced 293T cells with four lentiviral vectors (Azurite, GFP, Ametrine or mOrange as reporters) harboring shCD4 or shCD19 and tested the reporter-positive cells expansion over time (from day 4 to day 22). As shown in Figure 4F, we didn’t observe obvious difference of cell expansion between untransduced, single fluorescent reporter- transduced and four fluorescent reporter-transduced subsets at the given time point. Accordingly, we added a new paragraph as follows “The aim of this study was to develop a novel multiplex RNAi assay. We chose shRNAmir but not conventional shRNA in this assay because shRNAmir displayed minimal cytotoxicity. Indeed, our experimental data showed the co-transduction of 293T cells with four viruses carrying a neutral shRNAmir did not significantly alter the four fluorescent reporters-expressing cell expansion after 22 days of culture (Fig 4F). Since the multiple shRNAmirs-expressing cells always account for a small portion of whole cell population (<10% in this experiment), like low efficiency transduction of a single shRNAmir, it is very likely that they express each shRNAmir at single copy level. So, their total shRNAmir level will not be super abundant. Given the advantage of shRNAmir over conventional shRNA in maintaining cell homeostasis, multiple shRNAmir transduction might not be problematic.”

## 1st Revision - Editorial Decision 18th Jan 2022

Dear Dr. Wang

Thank you for the submission of your revised manuscript to EMBO reports. We have now received the full set of referee reports that is copied below.

As you will see, all referees are very positive about the study and request only minor changes to clarify text and figures. Please address the remaining concerns from referee 2.

From the editorial side, there are also a few things that we need before we can proceed with the official acceptance of your study.

- Author contributions: Please use abbreviations instead of the full names.

- Reference list: Please only list the first 10 authors, followed by et al.

- Please note that our editorial policies do not permit "Data not shown". You currently refer to such data on page 20 (figure legends). Please either include the relevant data to support your statement or remove it.

- Please add callouts to the panels of Fig. EV3 panel wherever appropriate.

- The first line (heading) of Table 2 appears strangely formatted (spacing between individual letters). Please double-check.

- Our production/data editors have asked you to clarify several points in the figure legends (see attached document). Please incorporate these changes in the attached word document and return the revised file with tracked changes with your final manuscript submission. I have also taken the liberty to make some changes to the title (may not exceed 100 characters) and abstract (should be written in present tense). Could you please review these?

- Please also add a header for the Expanded View Figure legends

- During our routine image and figure analysis we noted that all Western blots have rather high contrast settings. Please reduce the contrast and I also recommend providing the source data for the Western blots.

- Please note that all corresponding authors are required to supply an ORCID ID for their name upon submission of a revised manuscript (<https://orcid.org/>). This information is currently missing for Prof. Luo.
Please find instructions on how to link your ORCID ID to your account in our manuscript tracking system in our Author guidelines (<https://www.embopress.org/page/journal/14693178/authorguide#authorshipguidelines>)

- Finally, EMBO reports papers are accompanied online by A) a short (1-2 sentences) summary of the findings and their significance, B) 2-3 bullet points highlighting key results and C) a synopsis image that is 550x200-600 pixels large (width x height) in .png format. You can either show a model or key data in the synopsis image. Please note that the size is rather small and that text needs to be readable at the final size. Please send us this information along with the revised manuscript.

We look forward to seeing a final version of your manuscript as soon as possible.

With kind regards,

Martina Rembold, PhD
Senior Editor
EMBO reports

********************

Referee #1:

The authors have adequately addressed the concerns relating to the prior submission.

Referee #2:

The authors have performed new experiments and answered most of the raised points. There are two issues that remain to be resolved before publication:

1) The manuscript, especially the newly inserted parts should be thoroughly proofread and corrected by a native speaker.

2) Issue 4.2 Results: "miR-AB shows outstanding RNAi efficiency in vitro"
The authors addressed my point by showing that the shRNAmiRs Pten.1524 and Pten.932 have equal activity when expressed from miR-E and miR-AB. In addition, the show a GFP-reporter assay with three different shRNAs expressed from the two different backbones and claim "to show transduction efficiency and RNAi efficiency simultaneously." However, this is not true, what is shown in Fig. 2F is the transduction rate of the reporter. I would recommend to display the data in a 2D FACS plots with the GFP-reporter on the x-axis and the fluorescent protein coupled to the shRNA on the y-axis. In this way the knockdown and the transduction rate with the shRNA can be assessed. Note: the plot in Fig. 2F can be kept, but the 2D FACS plots as suggested should be included in the supplement.

1) The manuscript, especially the newly inserted parts should be thoroughly proofread and corrected by a native speaker.

**Response:** Thank you for pointing out the mistakes. We corrected as you suggested.

2) Issue 4.2 Results: "miR-AB shows outstanding RNAi efficiency in vitro"

The authors addressed my point by showing that the shRNAmiRs Pten.1524 and Pten.932 have equal activity when expressed from miR-E and miR-AB. In addition, the show a GFP-reporter assay with three different shRNAs expressed from the two different backbones and claim "to show transduction efficiency and RNAi efficiency simultaneously." However, this is not true, what is shown in Fig. 2F is the transduction rate of the reporter. I would recommend to display the data in a 2D FACS plots with the GFP-reporter on the x-axis and the fluorescent protein coupled to the shRNA on the y-axis. In this way the knockdown and the transduction rate with the shRNA can be assessed. Note: the plot in Fig. 2F can be kept, but the 2D FACS plots as suggested should be included in the supplement.

**Response:** The GFP reporter assay did show transduction efficiency and RNAi efficiency simultaneously. Our histograms give two information. One is the frequency distribution that indicates the transduction efficiency and the other is the MFI of a population (the location of a population on the X-axis). In this assay, there is no fluorescent protein coupled to the shRNA. The GFP-specific shRNAmir and GFP reporter are expressed from the same vector. To clarify, we revised the text as follows: **To this end, we cloned three GFP-specific shRNAmirs into the miR-AB or miR-E backbone in a lentiviral vector with a GFP reporter and analyzed their RNAi potency after transduction of MC38 or 293T cells.**

We also made a figure as follows. The figure shows the data in 2D FACS plots where the Y-axis is FSC-H. Both the 2D plots and histograms can show distribution frequency (transduction efficiency) nicely. However, the MFI info is easier to be read from the histogram.

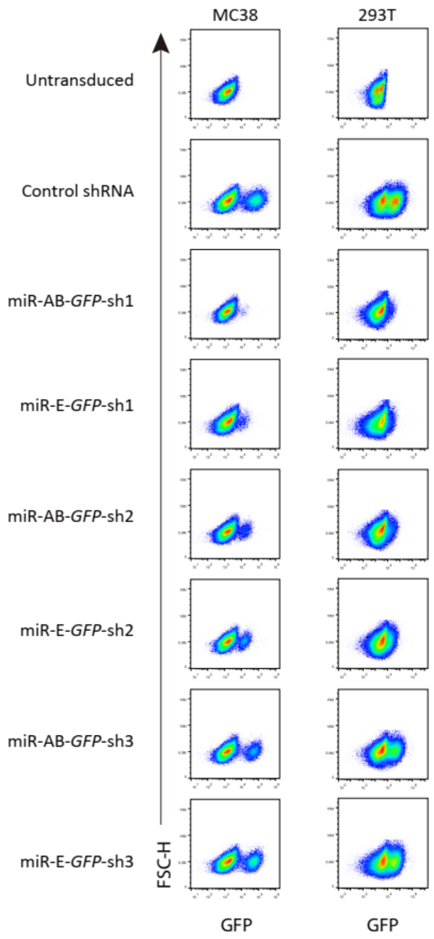

## 2nd Revision - Editorial Decision 30th Jan 2022

Dr. Dapeng Wang
Binzhou Medical College
Immunology
Guanhai Road 346, Laishan District
Yantai, Shandong 264003
China

Dear Dr. Wang,

I am very pleased to accept your manuscript for publication in the next available issue of EMBO reports. Thank you for your contribution to our journal.

At the end of this email I include important information about how to proceed. Please ensure that you take the time to read the information and complete and return the necessary forms to allow us to publish your manuscript as quickly as possible.

As part of the EMBO publication's Transparent Editorial Process, EMBO reports publishes online a Review Process File to accompany accepted manuscripts. As you are aware, this File will be published in conjunction with your paper and will include the referee reports, your point-by-point response and all pertinent correspondence relating to the manuscript.

If you do NOT want this File to be published, please inform the editorial office within 2 days, if you have not done so already, otherwise the File will be published by default [contact: emboreports@embo.org]. If you do opt out, the Review Process File link will point to the following statement: "No Review Process File is available with this article, as the authors have chosen not to make the review process public in this case."

Should you be planning a Press Release on your article, please get in contact with emboreports@wiley.com as early as possible, in order to coordinate publication and release dates.

Thank you again for your contribution to EMBO reports and congratulations on a successful publication. Please consider us again in the future for your most exciting work.

Yours sincerely,

Martina Rembold, PhD
Senior Editor
EMBO reports

*********************************************************************************

THINGS TO DO NOW:

You will receive proofs by e-mail approximately 2-3 weeks after all relevant files have been sent to our Production Office; you should return your corrections within 2 days of receiving the proofs.

Please inform us if there is likely to be any difficulty in reaching you at the above address at that time. Failure to meet our deadlines may result in a delay of publication, or publication without your corrections.

All further communications concerning your paper should quote reference number EMBOR-2021-53691V3 and be addressed to emboreports@wiley.com.

Should you be planning a Press Release on your article, please get in contact with emboreports@wiley.com as early as possible, in order to coordinate publication and release dates.

EMBO PRESS

YOU MUST COMPLETE ALL CELLS WITH A PINK BACKGROUND 🡣

PLEASE NOTE THAT THIS CHECKLIST WILL BE PUBLISHED ALONGSIDE YOUR PAPER

Corresponding Author Name: Dapeng Wang
Journal Submitted to: EMBO reports
Manuscript Number: EMBOR-2021-53691V1

## Reporting Checklist For Life Sciences Articles (Rev. June 2017)

This checklist is used to ensure good reporting standards and to improve the reproducibility of published results. These guidelines are consistent with the Principles and Guidelines for Reporting Preclinical Research issued by the NIH in 2014. Please follow the journal's authorship guidelines in preparing your manuscript.

### A- Figures
### 1. Data
### The data shown in figures should satisfy the following conditions:

- the data were obtained and processed according to the field's best practice and are presented to reflect the results of the experiments in an accurate and unbiased manner.
- figure panels include only data points, measurements or observations that can be compared to each other in a scientifically meaningful way.
- graphs include clearly labeled error bars for independent experiments and sample sizes. Unless justified, error bars should not be shown for technical replicates.
- if n< 5, the individual data points from each experiment should be plotted and any statistical test employed should be justified
- Source Data should be included to report the data underlying graphs. Please follow the guidelines set out in the author ship guidelines on Data Presentation.

### 2. Captions

### Each figure caption should contain the following information, for each panel where they are relevant:

- a specification of the experimental system investigated (eg cell line, species name).
- the assay(s) and method(s) used to carry out the reported observations and measurements
- an explicit mention of the biological and chemical entity(ies) that are being measured.
- an explicit mention of the biological and chemical entity(ies) that are altered/varied/perturbed in a controlled manner.
- the exact sample size (n) for each experimental group/condition, given as a number, not a range;
- a description of the sample collection allowing the reader to understand whether the samples represent technical or biological replicates (including how many animals, litters, cultures, etc.).
- a statement of how many times the experiment shown was independently replicated in the laboratory.
- definitions of statistical methods and measures:
  - common tests, such as t-test (please specify whether paired vs. unpaired), simple χ2 tests, Wilcoxon and Mann-Whitney tests, can be unambiguously identified by name only, but more complex techniques should be described in the methods section;
  - are tests one-sided or two-sided?
  - are there adjustments for multiple comparisons?
  - exact statistical test results, e.g., P values = x but not P values < x;
  - definition of 'center values' as median or average;
  - definition of error bars as s.d. or s.e.m.

Any descriptions too long for the figure legend should be included in the methods section and/or with the source data.

**In the pink boxes below, please ensure that the answers to the following questions are reported in the manuscript itself. Every question should be answered. If the question is not relevant to your research, please write NA (non applicable). We encourage you to include a specific subsection in the methods section for statistics, reagents, animal models and human subjects.**

## USEFUL LINKS FOR COMPLETING THIS FORM

http://www.antibodypedia.com
http://1degreebio.org
http://www.equator-network.org/reporting-guidelines/improving-bioscience-research-repor
http://grants.nih.gov/grants/olaw/olaw.htm
http://www.mrc.ac.uk/Ourresearch/Ethicsresearchguidance/Useofanimals/index.htm
http://ClinicalTrials.gov
http://www.consort-statement.org
http://www.consort-statement.org/checklists/view/32-consort/66-title
http://www.equator-network.org/reporting-guidelines/reporting-recommendations-for-tumo
http://datadryad.org
http://figshare.com
http://www.ncbi.nlm.nih.gov/gap
http://www.ebi.ac.uk/ega
http://biomodels.net/
http://biomodels.net/miriam/
http://jjj.biochem.sun.ac.za
https://osp.od.nih.gov/biosafety-biosecurity-and-emerging-biotechnology/
http://www.selectagents.gov/

### B- Statistics and general methods

Please fill out these boxes 🡣 (Do not worry if you cannot see all your text once you press return)

| | |
|---|---|
| 1.a. How was the sample size chosen to ensure adequate power to detect a pre-specified effect size? | Sample size was chosen according to most relevant studies. |
| 1.b. For animal studies, include a statement about sample size estimate even if no statistical methods were used. | For animal experiments, five age- and sex-matched mice was used for each group. |
| 2. Describe inclusion/exclusion criteria if samples or animals were excluded from the analysis. Were the criteria pre-established? | No samples or animals were excluded from the analysis in this study. |
| 3. Were any steps taken to minimize the effects of subjective bias when allocating animals/samples to treatment (e.g. randomization procedure)? If yes, please describe. | No. |
| For animal studies, include a statement about randomization even if no randomization was used. | Mice were randomly grouped in this study. |
| 4.a. Were any steps taken to minimize the effects of subjective bias during group allocation or/and when assessing results (e.g. blinding of the investigator)? If yes please describe. | No. |
| 4.b. For animal studies, include a statement about blinding even if no blinding was done | Data collection and analysis for animal experiments were blinded. |
| 5. For every figure, are statistical tests justified as appropriate? | Yes. |
| Do the data meet the assumptions of the tests (e.g., normal distribution)? Describe any methods used to assess it. | Yes. Unpaired Student T test was used in this study. |
| Is there an estimate of variation within each group of data? | We included standard deviation (s.d.) in the experiments. |

| | |
|---|---|
| Is the variance similar between the groups that are being statistically compared? | Yes. |

### C- Reagents

| | |
|---|---|
| 6. To show that antibodies were profiled for use in the system under study (assay and species), provide a citation, catalog number and/or clone number, supplementary information or reference to an antibody validation profile. e.g., Antibodypedia (see link list at top right), 1DegreeBio (see link list at top right). | All antibodies used in this study were purchased from antibody companies where the antibody validation was done. The catalog number of each antibody used in this study was provided in the Materials and Methods section. |
| 7. Identify the source of cell lines and report if they were recently authenticated (e.g., by STR profiling) and tested for mycoplasma contamination. | All cell lines were directly or indirectly from a cell line deposition institute, such as ATCC or Peking Union Medical College. |

* for all hyperlinks, please see the table at the top right of the document

### D- Animal Models

| | |
|---|---|
| 8. Report species, strain, gender, age of animals and genetic modification status where applicable. Please detail housing and husbandry conditions and the source of animals. | All details have been described in the Materials and Methods section. |
| 9. For experiments involving live vertebrates, include a statement of compliance with ethical regulations and identify the committee(s) approving the experiments. | "The Binzhou Medical University Animal Care and Use Committee approved all mouse experiments included in this work." has been included in the Materials and Methods section. |
| 10. We recommend consulting the ARRIVE guidelines (see link list at top right) (PLoS Biol. 8(6), e1000412, 2010) to ensure that other relevant aspects of animal studies are adequately reported. See author guidelines, under 'Reporting Guidelines'. See also: NIH (see link list at top right) and MRC (see link list at top right) recommendations. Please confirm compliance. | All relevant aspects of mice experiments in this study has been described. |

### E- Human Subjects

| | |
|---|---|
| 11. Identify the committee(s) approving the study protocol. | No human subjects were included in this study. |
| 12. Include a statement confirming that informed consent was obtained from all subjects and that the experiments conformed to the principles set out in the WMA Declaration of Helsinki and the Department of Health and Human Services Belmont Report. | NA. |
| 13. For publication of patient photos, include a statement confirming that consent to publish was obtained. | NA. |
| 14. Report any restrictions on the availability (and/or on the use) of human data or samples. | NA. |
| 15. Report the clinical trial registration number (at ClinicalTrials.gov or equivalent), where applicable. | NA. |
| 16. For phase II and III randomized controlled trials, please refer to the CONSORT flow diagram (see link list at top right) and submit the CONSORT checklist (see link list at top right) with your submission. See author guidelines, under 'Reporting Guidelines'. Please confirm you have submitted this list. | NA. |
| 17. For tumor marker prognostic studies, we recommend that you follow the REMARK reporting guidelines (see link list at top right). See author guidelines, under 'Reporting Guidelines'. Please confirm you have followed these guidelines. | NA. |

### F- Data Accessibility

| | |
|---|---|
| 18: Provide a "Data Availability" section at the end of the Materials & Methods, listing the accession codes for data generated in this study and deposited in a public database (e.g. RNA-Seq data: Gene Expression Omnibus GSE39462, Proteomics data: PRIDE PXD000208 etc.) Please refer to our author guidelines for 'Data Deposition'.<br><br>Data deposition in a public repository is mandatory for:<br>a. Protein, DNA and RNA sequences<br>b. Macromolecular structures<br>c. Crystallographic data for small molecules<br>d. Functional genomics data<br>e. Proteomics and molecular interactions | No primary datasets have been generated and deposited. |
| 19. Deposition is strongly recommended for any datasets that are central and integral to the study; please consider the journal's data policy. If no structured public repository exists for a given data type, we encourage the provision of datasets in the manuscript as a Supplementary Document (see author guidelines under 'Expanded View' or in unstructured repositories such as Dryad (see link list at top right) or Figshare (see link list at top right). | NA |
| 20. Access to human clinical and genomic datasets should be provided with as few restrictions as possible while respecting ethical obligations to the patients and relevant medical and legal issues. If practically possible and compatible with the individual consent agreement used in the study, such data should be deposited in one of the major public access-controlled repositories such as dbGAP (see link list at top right) or EGA (see link list at top right). | NA |
| 21. Computational models that are central and integral to a study should be shared without restrictions and provided in a machine-readable form. The relevant accession numbers or links should be provided. When possible, standardized format (SBML, CellML) should be used instead of scripts (e.g. MATLAB). Authors are strongly encouraged to follow the MIRIAM guidelines (see link list at top right) and deposit their model in a public database such as Biomodels (see link list at top right) or JWS Online (see link list at top right). If computer source code is provided with the paper, it should be deposited in a public repository or included in supplementary information. | NA |

### G- Dual use research of concern

| | |
|---|---|
| 22. Could your study fall under dual use research restrictions? Please check biosecurity documents (see link list at top right) and list of select agents and toxins (APHIS/CDC) (see link list at top right). According to our biosecurity guidelines, provide a statement only if it could. | No. |
